# Survey of the binding preferences of RNA-binding proteins to RNA editing events

**DOI:** 10.1186/s13059-022-02741-8

**Published:** 2022-08-04

**Authors:** Xiaolin Hu, Qin Zou, Li Yao, Xuerui Yang

**Affiliations:** 1 School of Public Health, Shanghai Jiao Tong University School of Medicine, Shanghai, 200025 China; 2 Key Laboratory of Molecular Medicine and Biotherapy, School of Life Science, Beijing Institute of Technology, Beijing, 100081 China; 3 MOE Key Laboratory of Bioinformatics, School of Life Sciences, Tsinghua University, Beijing, 100084 China; 4 Center for Synthetic & Systems Biology, Tsinghua University, Beijing, 100084 China

## Abstract

**Background:**

Adenosine-to-inosine (A-to-I) editing is an important RNA posttranscriptional process related to a multitude of cellular and molecular activities. However, systematic characterizations of whether and how the events of RNA editing are associated with the binding preferences of RNA sequences to RNA-binding proteins (RBPs) are still lacking.

**Results:**

With the RNA-seq and RBP eCLIP-seq datasets from the ENCODE project, we quantitatively survey the binding preferences of 150 RBPs to RNA editing events, followed by experimental validations. Such analyses of the RBP-associated RNA editing at nucleotide resolution and genome-wide scale shed light on the involvement of RBPs specifically in RNA editing-related processes, such as RNA splicing, RNA secondary structures, RNA decay, and other posttranscriptional processes.

**Conclusions:**

These results highlight the relevance of RNA editing in the functions of many RBPs and therefore serve as a resource for further characterization of the functional associations between various RNA editing events and RBPs.

**Supplementary Information:**

The online version contains supplementary material available at 10.1186/s13059-022-02741-8.

## Background

RNA editing via nucleobase deamination is an important procedure of posttranscriptional RNA processing that results in adenosine-to-inosine (A-to-I) or cytidine-to-uridine (C-to-U) conversion [1]. In mammalian cells, A-to-I editing, which is the predominant type, is carried out by ADAR (adenosine deaminases that act on RNA) family proteins [2, 3], and C-to-U editing is carried out by APOBEC1 with the cofactor A1CF [4]. RNA editing can take place in both prespliced and mature RNAs [5]. While RNA editing sites have been found throughout the transcriptome, they are mostly enriched in Alu element sequences in the 3′ untranslated regions (3′ UTRs) and introns [6].

Due to changes in the RNA sequence, RNA editing results in a multitude of consequences. Editing in mRNA-coding regions could lead to increased protein variability due to translation of the altered codons [7]. Another major impact of RNA editing is alternative splicing [5, 8]. For example, ADAR2 in rats can edit its own pre-mRNA in the 4th intron, generating a new 3′ splicing acceptor. This leads to alternative splicing of the pre-mRNA and introduction of an early stop codon, which serves as a machinery of negative feedback in controlling the level of ADAR2 [9]. RNA editing could also result in changes in RNA secondary structures, including both the formation and disassembly of double-stranded RNA structures [10, 11]. This has been shown to be related to the escape of the dsRNA-induced immune response in the cytoplasm [12].

Approximately 1000–2000 human proteins have been annotated as RNA-binding proteins (RBPs) [13], most of which show strong sequence or structure selectivity when binding to their target RNA [14]. Due to specific RNA-binding preferences, many RBPs are involved in various RNA-related biological processes [13], including transcriptional [15] and posttranscriptional processing [16], translation regulation [17], RNA decay [18], etc. Comprehensive and precise allocations of the binding target sequences of RBPs in the transcriptome are highly valuable for the elucidation of their molecular functions. A series of high-throughput methods, such as HITS-CLIP [19], PAR-CLIP [20], iCLIP [21], and eCLIP [22], have been developed for such purposes. Most of these methods are based on the immunoprecipitation of RBPs, followed by RNA profiling with next-generation sequencing. To date, many RBPs have been surveyed with at least one of these CLIP-based methods, producing insightful data for characterizing the molecular functions of the RBPs. For example, the ENCODE project has profiled approximately 150 RBPs with eCLIP in two cell lines, HepG2 and K562 [23], which is currently the most comprehensive source of RBP CLIP data.

Binding of an RBP to the target RNA could be dependent on the RNA sequence, secondary and higher order structures, and potentially other third-party molecules [14]. Many of these features could be affected by RNA editing events. However, it has not been systematically studied whether and how editing levels at specific sites affect RBP binding at the genome-wide scale. In the present study, based on the RNA-seq and the RBP eCLIP data generated by the ENCODE project, we performed quantitative assessments of the RNA editing levels in the RBP eCLIP data while taking the regular RNA-seq data as the background. Such analyses yielded comprehensive evaluations of the RBP’s preferences for edited or unedited RNA at the resolution of single editing sites. The study showed that some RBPs indeed have strong binding preferences for edited or unedited RNA at the global scale or in a site-specific manner. Analyses of these binding patterns shed light on the involvement of RBPs specifically in RNA editing-related processes, such as RNA splicing, RNA secondary structures, RNA decay, and potentially other posttranscriptional processes. In summary, the present study highlights the relevance of RNA editing in the functions of many RBPs and therefore serves as a resource for further characterization of the functional associations between the various RNA editing events and RBPs.

## Results

### Profiles of RNA editing events from RNA-seq data

The ENCODE project has generated RNA deep sequencing data for different fractions of HepG2 and K562 cells [23]. For the same two cell lines, ENCODE also performed comprehensive profiling of the RBP-bound RNA with eCLIP, covering 150 RBPs. Given the high data quality, consistent experimental procedures, and the largest scale of its kind, this collection of data allows us to systematically assess RNA editing events in the RBP-associated transcriptome by using the cell transcriptome as the background. The RBP-associated transcriptomes profiled by eCLIP were obtained with whole cell extracts, but the RBPs themselves usually have subcellular location preferences, such as cytosol or nucleus (details of the RBP locations are listed in Additional file 2: Table S1) [24]. Therefore, we first profiled the RNA editing events in different cell fractions (nucleus, cytosol, and whole cell) with the total RNA-seq data of the fractionated HepG2 and K562 cell extracts from ENCODE.

**Fig. 1:**
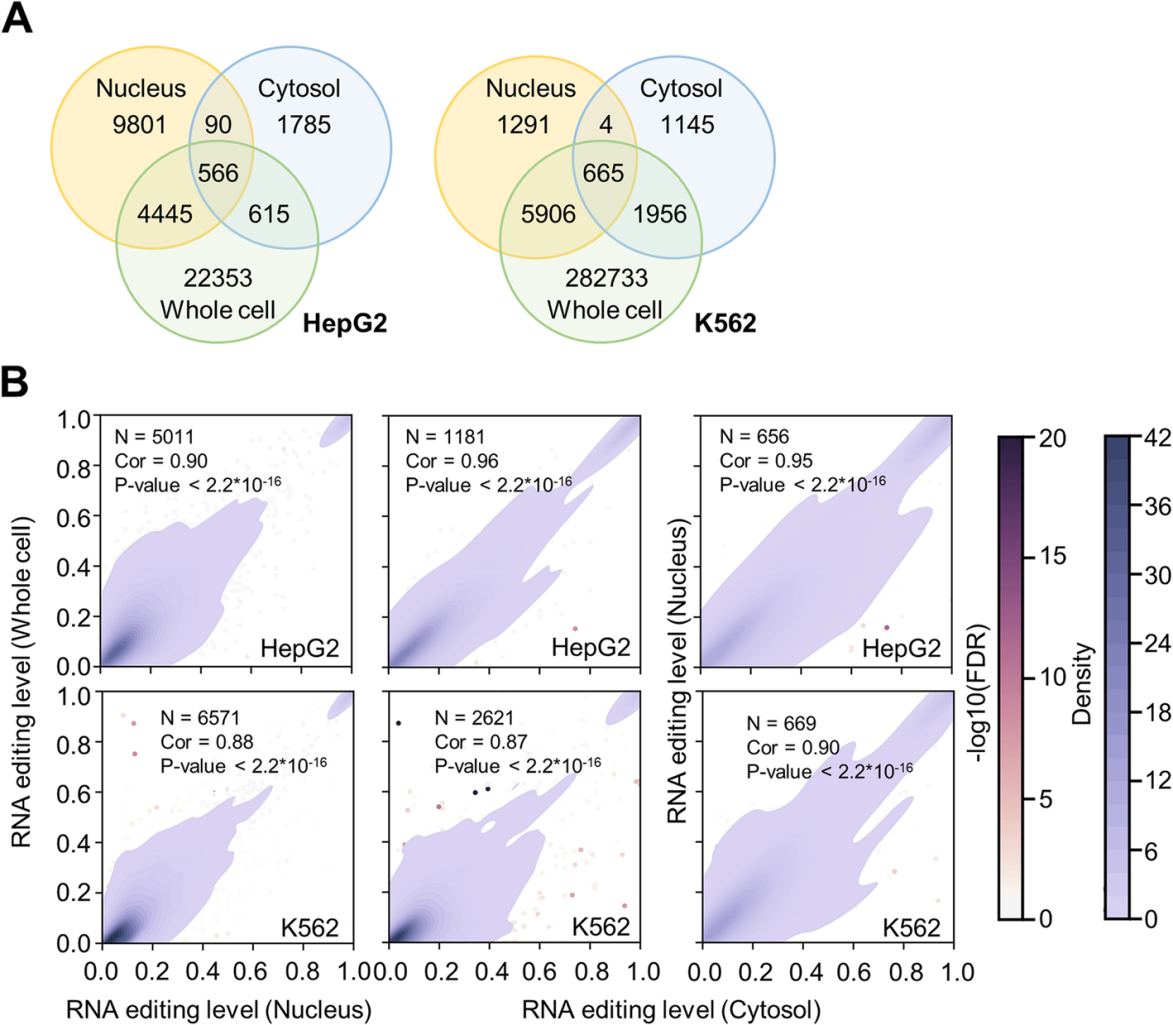
Profiles of A-to-I RNA editing events in HepG2 and K562 cells. **A** The overlaps among the A-to-I RNA editing events detected in different fractions of HepG2 and K562 cells. **B** Pearson correlations of the RNA editing levels for the sites detected simultaneously in different cellular fractions. Left: whole cell vs. nucleus; middle: whole cell vs. cytosol; right: nucleus vs. cytosol. Each dot represents an editing site, and the total number in each plot is provided (*N*). Dot density is colored with blue gradient. The editing sites showing significantly different editing levels between the fractions are colored according to the FDR values

As expected, most of the A-to-I RNA editing events identified from the RNA-seq data above were found in the Alu elements (Additional file 1: Fig. S1A). The editing sites showed a weak motif of U/A/C[A]S (‘S’ for strong G) (Additional file 1: Fig. S1B), which is highly consistent with previous reports [25–27]. The vast majority of the RNA editing events were only detected in the RNA-seq data of the whole cell fraction (Fig. 1A), and this is at least partly due to the much higher sequencing depth for the whole cell fraction samples (Additional file 1: Fig. S1C, D, Additional file 3: Table S2). Next, small proportions of the A-to-I RNA editing events were detected in both the nuclear and cytosolic fractions, and the majority of the RNA editing events were detected in the nucleus or cytosol only (Fig. 1A). This is consistent with previous understandings about the subcellular preferences of the different RNA editing events, i.e., the RNA editing events taking place in the introns are spliced out and retained in the nucleus, while the editing events carried out by cytosolic ADAR P150 should be mostly located in the cytosol [28].

For the relatively small numbers of A-to-I RNA editing events detected in two out of the three fractions (whole cell, nucleus, and cytosol), their editing levels were generally consistent across different fractions (Pearson correlation higher than 0.87 in K562 and 0.90 in HepG2, *P*-value smaller than 2.2 × 10^−16^, Fig. 1B), except for a few sites with significantly differential editing levels (Fig. 1B). These sites were removed for further analyses. Finally, HepG2 and K562 cells shared small proportions of RNA editing events (Additional file 1: Fig. S2A), which is in line with the previously reported strong cell type specificity of RNA editing [29–32]. However, in general, the overlapping editing sites share relatively similar editing levels in the two cell lines, except for some outliers (Additional file 1: Fig. S2B).

### Profiles of RBP-binding RNA editing sites

Many of the RNA editing sites identified above are located within the binding target sequences of at least one RBP, which were revealed by the eCLIP data (Additional file 4: Table S3). Some RBPs, such as HNRNPC, ILF3, and UPF1, showed high frequencies of binding to the regions with RNA editing sites in both HepG2 and K562 cells (Additional file 1: Fig. S3A, B). Although some of these RBPs have been reported to be related to the regulation of RNA editing, such as ILF3 [30, 33], many others have not been associated with RNA editing with a clear machinery. Therefore, it is worth further evaluating the potential associations between these RBPs and RNA editing by first checking their binding preferences for edited or unedited RNA sequences.

**Fig. 2:**
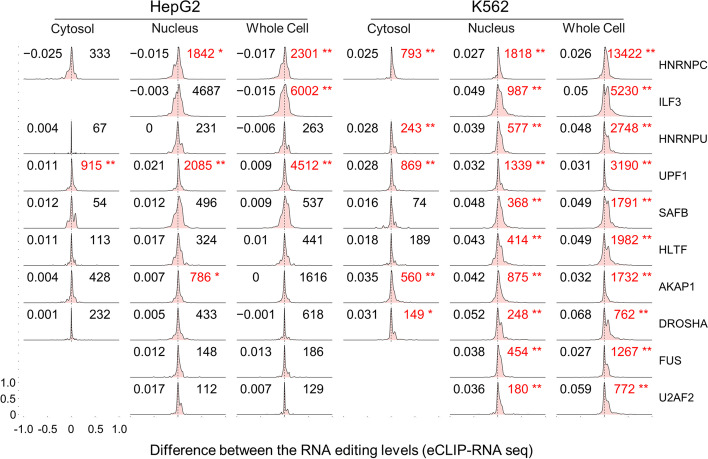
Overview of the editing-level differences in RBP eCLIP and RNA-seq data. Distributions of the differential editing levels of the editing sites in each RBP eCLIP and the RNA-seq data. Numbers of A-to-I RNA editing events detected in both the RBP eCLIP and RNA-seq data are provided in the top right corner of each subplot. *P*-values were calculated by paired Wilcoxon signed tests. The significant tests, with a cutoff of 10^−5^, are marked in red. **P-value less than 10^−5^; ***less than 10^−10^. The median differential editing level of all the editing sites in each subplot is provided in the top left corner

Specifically, for the editing sites that reside in the RBP-binding regions, we examined their editing levels in the RBP-specific eCLIP data. Comparison between the RNA editing levels in the RBP eCLIP data and those in the total cell or the fractionated RNA-seq data should provide assessments of the binding preferences of the RBP to the RNA editing sites. Note that for the RBPs whose intracellular localizations were clearly defined [24], we only used the RNA editing profile of the corresponding cell fraction as the background. The differences between RBP-bound RNA editing profiles and the background RNA editing profiles in HepG2 or K562 cells were quantitatively evaluated with paired Wilcoxon tests (614 comparisons in total, shown in Additional file 5: Table S4). A total of 207 out of the 614 comparisons exhibited significant differences (*P*-value < 10^−5^) in the RBP-specific eCLIP data (examples shown in Fig. 2, Additional file 1: Fig. S4, and the full data in Additional file 5: Table S4 and Additional file 6).

The results above showed that for a number of RBPs, the editing levels of many sites in the eCLIP data were markedly different than the RNA editing profiles in the RNA-seq data. HNRNPC, ILF3, HNRNPU, UPF1, and SAFB were the top 5 RBPs with the most significant overall difference between the RBP-bound RNA editing levels and the background RNA editing profile (Fig. 2). Notably, some RBPs showed overwhelming preferences for either the edited or unedited RNA sequences, such as ILF3 and HNRNPU in K562 cells and SAFB in both cell lines (Fig. 2). However, for many other RBPs, their preferences toward edited or unedited RNA vary across different RNA editing sites. This prompted us to further examine the RBP-binding preferences for specific RNA editing events.

### Preferences of RBPs to specific RNA editing events

Here, we developed a pipeline named *RBPed* to explore the potential preferences of RBPs to specific A-to-I edited or unedited RBP-binding sequences by comparing the editing levels in the eCLIP and total RNA-seq data. Specifically, Fisher’s exact test was introduced to evaluate the statistical significance of the differences in RNA editing levels between eCLIP-seq and RNA-seq, which takes into account the stochastic noise of the sequencing reads, especially for low-coverage regions [33] (details in the “Methods” section). Finally, for each RBP, with a statistical cutoff of adjusted *P*-value < 0.05 and an editing level difference larger than 0.1, our analysis identified editing favoring (editing level in eCLIP higher than that in RNA-seq) and disfavoring (editing level in eCLIP lower than that in RNA-seq) RNA sequences for each RBP (Additional file 7: Table S5). The majority of the RBP-associated RNA editing sites were identified by using the whole-cell RNA-seq data and the eCLIP-seq datasets, which can be partly attributed to the high sequencing depth and more editing sites in the whole-cell RNA-seq data (Additional file 1: Fig. S5). Furthermore, 50–70% of the RBP-editing associations identified with the cytosolic or nuclear fraction RNA-seq data were also found with the whole-cell RNA-seq data (Additional file 1: Fig. S5). Therefore, the subsequent analyses were done with the sites identified from the whole-cell data.

**Fig. 3:**
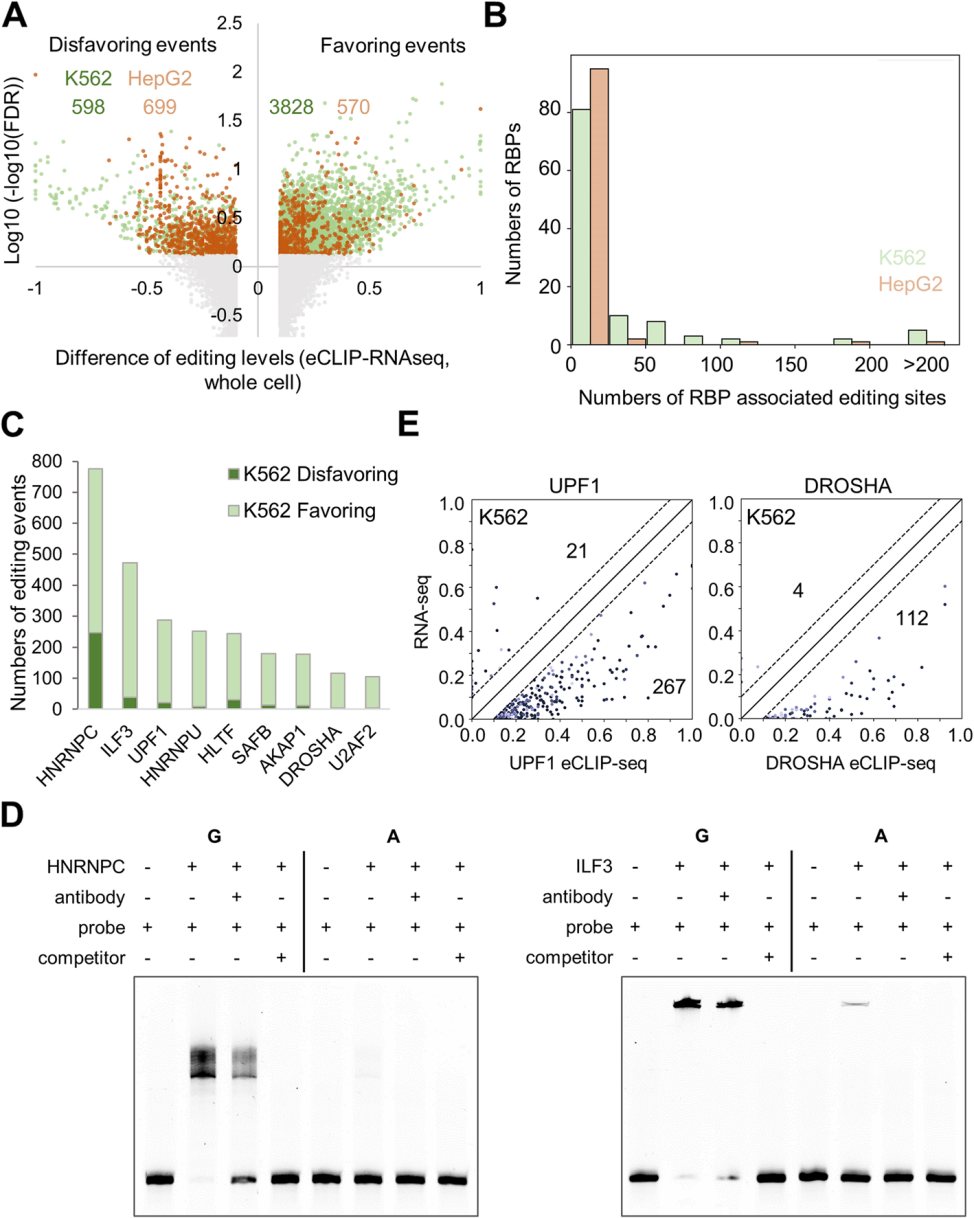
Preferences of RBPs to specific RNA editing events. **A** Statistical analysis of the differential RNA editing levels in eCLIP vs. whole-cell RNA-seq in K562 (green) and HepG2 (brown) cells. Each dot represents a single RNA editing site. The *X*-axis shows the difference in the editing levels, and the *Y*-axis shows the significance level. **B** Overall statistics of the numbers of RBP-associated editing sites for the 150 RBPs in K562 and HepG2 cells. **C** The numbers of RNA editing events favored or disfavored by the top 9 RBPs with the largest numbers of RBP-associated editing sites. **D** EMSA assays showing the shifted RNA bands upon preincubation with the two proteins HNRNPC and ILF3, respectively. **E** Scatter plots of the UPF1- and DROSHA-associated editing sites, showing their editing levels in the eCLIP data (*X*-axis) and in the whole-cell RNA-seq data (*Y*-axis). The numbers of sites falling in each triangle domain are provided on the plots

A total of 5695 RNA editing sites exhibited differential binding preferences of their edited and unedited forms by at least one RBP in the whole cell fraction (Fig. 3A, Additional file 7: Table S5). Among them are some RNA editing sites previously identified to be physiologically relevant in the development of cancer and other diseases [31] (Additional file 8: Table S6), whereas the underlying mechanisms are unclear. Functions of the RBPs associated with these RNA editing sites could shed light on how these editing events are related to biological activities and disease development.

Most of the 150 RBPs showed significantly favorable or unfavorable binding to a few RNA editing sites (Fig. 3B, Additional file 1: Fig. S6). Note that in general, there were more editing sites significantly associated with RBPs in K562 cells than in HepG2 cells (Fig. 3A, Additional file 1: Fig. S6). This again is mainly attributed to the much deeper sequencing and more biological replicates in K562 (Additional file 1: Fig. S1C, D). For a particular RBP, it is usually associated with largely different sets of RNA editing sites in the two cell lines. However, it is rarely seen that the same editing event shows opposite binding preferences for an RBP in the two cell lines (only 10 out of 5695 pairs of RBP-editing sites), i.e., favorable in one cell but disfavorable in the other.

Multiple factors could have contributed to the difference between the RBP-associated editing sites in the two cell lines, such as the different transcriptome landscapes and the RNA editing profiles between the two cell lines [30], different RNA-seq and eCLIP-seq coverages, and potentially the differential molecular functions of the RBPs in the two cell lines [30]. In fact, the candidate sites as input of the analysis pipeline were largely different between the two cell lines (Additional file 1: Fig. S2). In addition, it is likely that many of the RBP-associated RNA editing events have been missing (false negatives) due to the stringent filter criteria and the conserved pipeline that we implemented to reduce false discoveries.

The RNA editing sites tend to be enriched in clusters, but in the RBP-binding regions harboring multiple editing sites that are significantly associated with the RBP, these sites overwhelmingly show the same patterns of RBP-binding preference, i.e., editing events being favored or unfavored by the RBP (Additional file 1: Fig. S7). Very few of the RBP-binding regions actually harbored multiple sites with opposite binding preferences by the RBPs (Additional file 1: Fig. S7).

The 9 RBPs HNRNPC, ILF3, UPF1, HNRNPU, HLTF, SAFB, AKAP1, DROSHA, and U2AF2 exhibited strong preferences for edited or unedited RNA sequences for the largest numbers of RNA editing sites (Fig. 3C, Additional file 1: Fig. S6). Some of them have been well recognized to be closely related to RNA editing. For example, HNRNPC regulates per-mRNA splicing, mostly by suppressing exonization of the Alu introns [34–36], where RNA editing is highly enriched [37]. ILF3 serves as a repressor of RNA editing in both K562 and HepG2 cells by interacting with ADAR1 and binding close to ADAR1’s target sequences [30, 33]. It has also been shown that ILF3 regulates circRNA biogenesis by binding to the regions near the highly edited Alu elements [37, 38]. As an example from our results above, we selected an RNA fragment of 32 nt, which showed more favorable binding by both HNRNPC and ILF3 upon its editing at a particular editing site. Electrophoretic mobility shift (EMSA) assays directly confirmed the preferable binding of these two RBPs to the edited RNA but not to the unedited form (Fig. 3D).

The association between the ADAR-mediated A-to-I RNA editing and the UPF1-mediated RNA degradation has been well established before [39]. Such an association was proposed to be a machinery of RNA surveillance mediated by ADAR1 A-to-I editing and UPF1-dependent RNA degradation. Indeed, UPF1 showed strong preferences for the edited RNA (Fig. 3E). Considering the previous reports and our observations here, we highly suspect that the editing level of the RNA sequence is at play in UPF1-mediated RNA processing, potentially RNA degradation.

DROSHA is well known for its role in miRNA biogenesis, and recent studies have also shown that knockdown of DROSHA reduces RNA editing in K562 cells, potentially via direct interaction with ADAR1 [30]. Furthermore, by interacting with DROSHA and the non-Alu regions harboring pri-miRNAs, ADAR1 enhanced mature miRNA production in HeLa cells [40]. Our analysis showed a strong bias of DROSHA toward the edited RNA sequences (Fig. 3E). We did not find a clear and simple connection between the DROSHA-mediated miRNA maturation and the DROSHA-associated RNA editing events, but it is worth further in-depth investigation in the future whether the binding preferences of DROSHA to specific RNA editing sites are related to its function in controlling miRNA maturation.

### RNA editing events associated with multiple RBPs

**Fig. 4:**
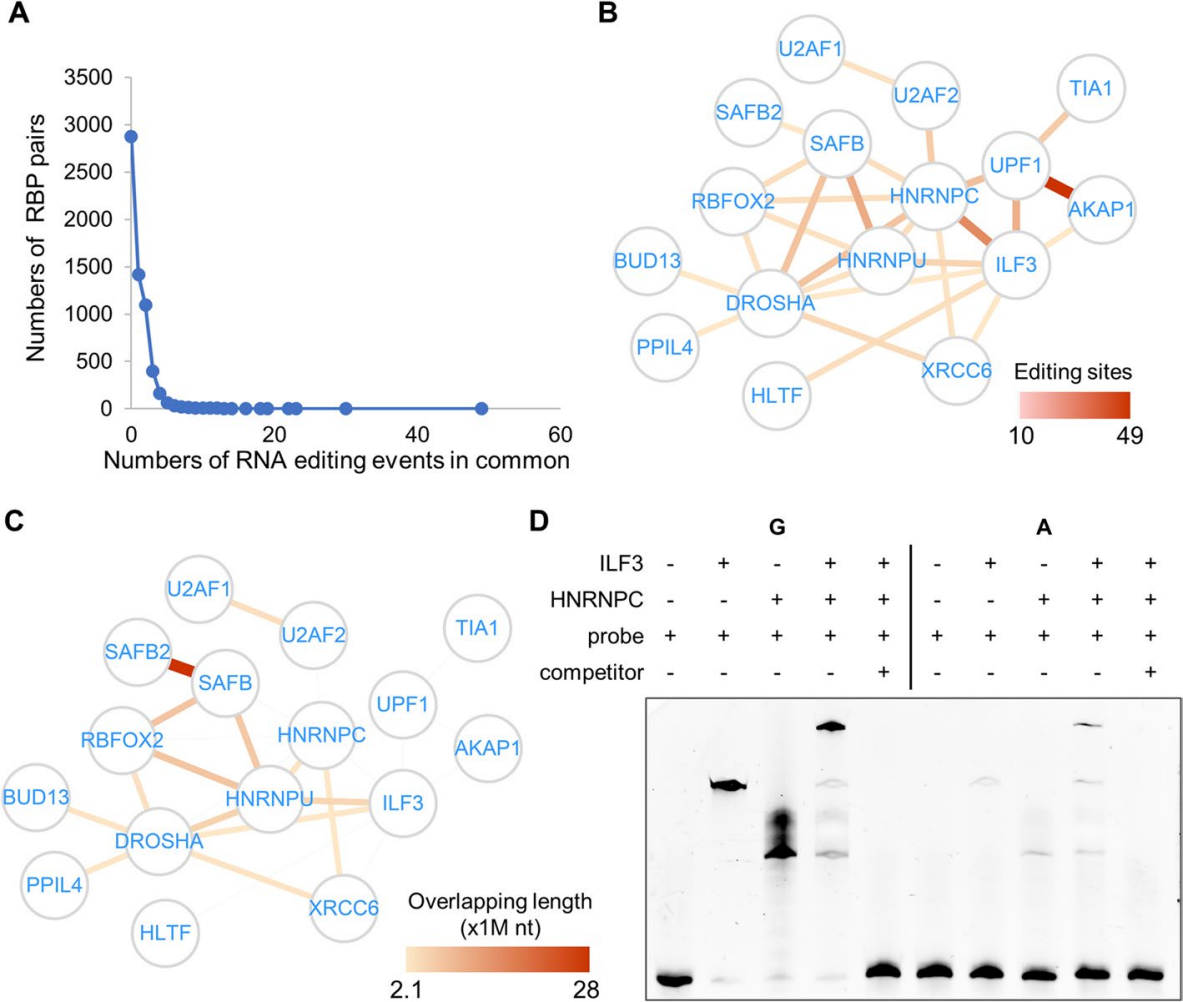
RBP interaction network based on RBP-associated RNA editing sites. **A** Numbers of RBP pairs (*Y*-axis) sharing the same groups of RNA editing events (*X*-axis). **B** RBP interaction network showing the overlapping RNA editing events associated with each pair of RBPs in K562 cells. The thickness of the edges indicates the number of shared editing sites. **C** RBP interaction network based on the lengths of the overlapping RBP-binding RNA regions. **D** EMSA assay showing the shifted RNA bands upon preincubation with the two proteins HNRNPC and ILF3 pooled together

Based on the results above, it is noted that some RNA editing events were associated with more than one RBP, especially in K562 (Fig. 4A). We therefore built an RBP interaction network as a summary of the overlapping RNA editing events associated with each pair of RBPs in K562 (Fig. 4B, Additional file 1: Fig. S8A). The interactions in the network were weighted by the numbers of the shared RNA editing events, based on the assumption that two RBPs preferably binding to the same set of edited RNA regions are likely to have functional associations mediated by RNA editing. Note that this RBP interaction map appears markedly different than the network simply based on the eCLIP peaks shared by RBP pairs (Fig. 4C, Additional file 1: Fig. S8A). Indeed, the similarities between the RBPs based on their overlapping RNA regions or the RBP-associated RNA editing sites showed very different patterns (Additional file 1: Fig. S8B). In addition, we used the network comparison test (NCT) [41] to evaluate the difference between the two networks. With 2000 permutations, the *P*-values of the global strength and network invariance were both estimated to be lower than 0.0005, thus confirming that the RBP interactions based on the associated RNA editing events are not simply due to the shared binding regions on the RNA molecules.

As discussed above for the EMSA results in Fig. 3D, an RNA fragment, upon its editing, showed more favorable binding by both HNRNPC and ILF3. Here, a new EMSA assay with the same RNA but the two RBPs pooled together further showed that HNRNPC and ILF3 indeed can bind to the same edited RNA sequence simultaneously, but not to the unedited form (Fig. 4D). This supports the concept of RNA editing events being associated with multiple RBPs.

In fact, 3 proteins, HNRNPC, UPF1, and ILF3, formed a small interaction module in the RBP network (Fig. 4B). Indeed, we previously showed that HNRNPC controls the splicing of Alu introns. Repression of HNRNPC resulted in Alu exonization, which triggered nonsense-mediated decay (NMD) and in turn gave rise to the accumulation of dsRNA enriched by Alu sequences [34]. This cascade of RNA posttranscriptional processing eventually led to the activation of the interferon response in breast cancer cells. During this process, UPF1 plays a critical role in mediating the NMD of Alu-enriched RNA and generating Alu-enriched dsRNA [34]. Given that RNA editing sites are enriched in Alu RNA, our results support the coordinated effects of HNRNPC and UPF1 in controlling Alu-originated endogenous dsRNA due to alternative splicing of the Alu introns. Interestingly, in the RBP interaction network inferred only based on the shared eCLIP peaks, the HNRNPC-UPF1 interaction was missing (Fig. 4C, Additional file 1: Fig. S8A). This illustrates the unique value of the shared RBP-associated RNA editing events in uncovering special RBP interactions.

Similarly, UPF1 and ILF3 showed a strong interaction based on their common preferences for RNA editing events (Fig. 4B), whereas such interaction was absent in the RBP network based purely on the eCLIP-seq data (Fig. 4C, Additional file 1: Fig. S8A). Recent studies have shown an interplay between UPF1 and ILF3 in regulating each other in hepatocellular carcinoma [42]. UPF1 binds to the 3′ UTR of ILF3, resulting in alternation of the polyadenylation, whereas ILF3 binds near the alternative donor site of UPF1 exon 7 and likely regulates the splicing of this exon [42]. In addition, another study showed that the NF45-NF90 (ILF2-ILF3) complex competes with Stau1/2-UPF1 for binding to the mitotic mRNA’s 3′ UTR, thereby promoting the expression of mitotic mRNA and mitotic fitness [43].

Finally, it was noted that some key proteins involved in RNA splicing, RNA stability and decay, miRNA processing, and RNA export exhibited enriched connections in the RNA editing-associated RBP interaction network but not in the network based on the shared eCLIP peaks (Additional file 1: Fig. S9, S10). Further investigations on these RBP interactions could shed more light on how RBP-associated RNA editing events might be involved in the regulation of these key RNA processes.

### RNA secondary structure changes related to RNA editing

The binding affinities of RBPs to their target RNA sequence depend on the sequence itself as well as the RNA secondary structures [14, 44, 45]. Therefore, we aimed to determine whether the preferences of RBPs to edited or unedited RNA could be attributed to RNA secondary structure differences upon RNA editing.

**Fig. 5:**
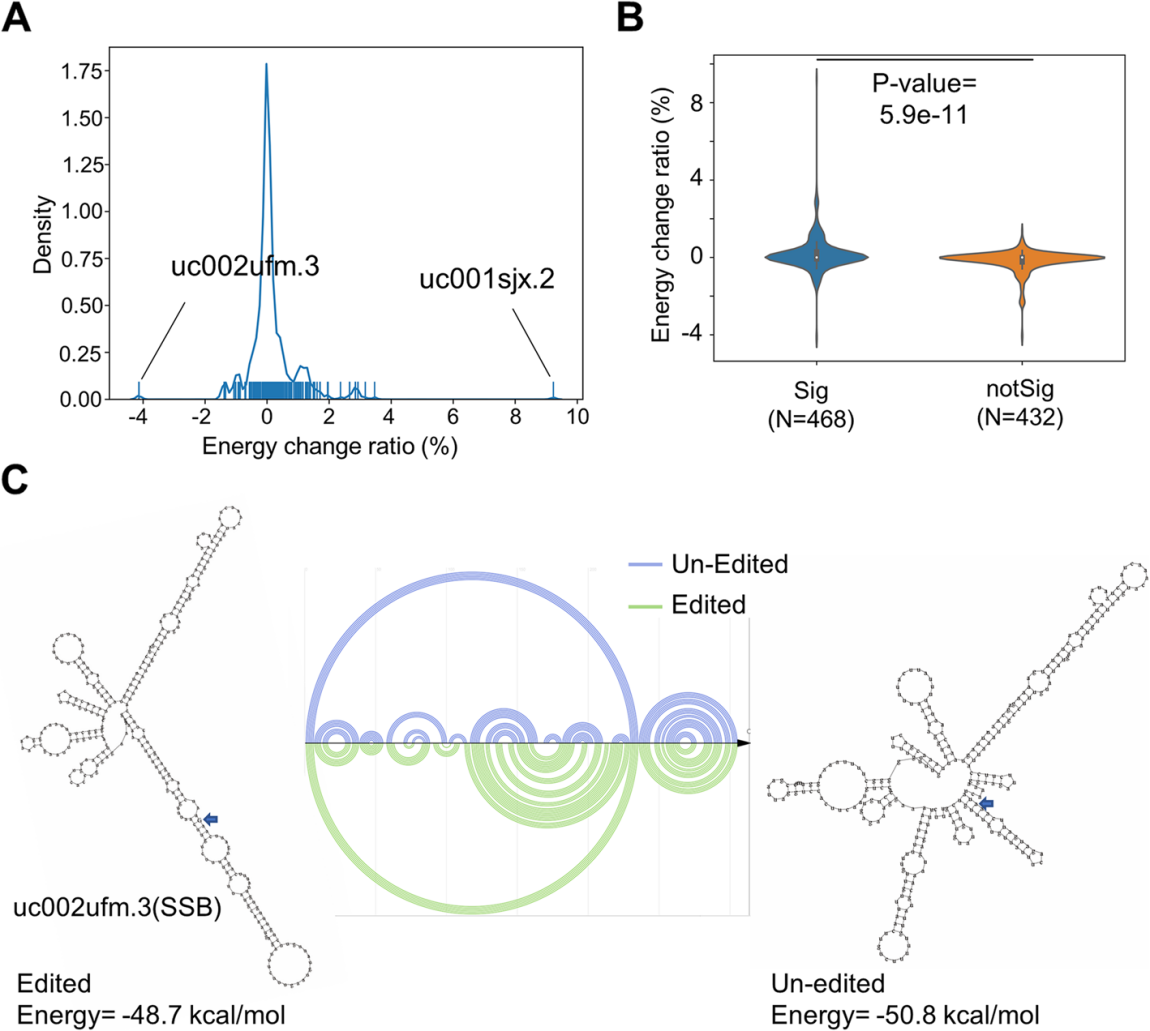
Secondary structure changes upon RBP-associated RNA editing in the 3′ UTR. **A** The predicted minimum free energy (MFE) difference of the 3′ UTR sequences harboring the RBP-associated RNA editing sites before and after RNA editing. The PARS-seq data of HepG2 cells upon ADAR1 knockdown were used as a constraint. **B** Comparison between the distribution of the MFE change shown in panel **A** for the 3′ UTR sequences harboring the RBP-associated RNA editing sites (Sig) and the distribution for the 3′ UTR sequences harboring the similar numbers of editing sites that fall in the RBP-bound RNA regions but its editing not significantly associated with the RBPs (notSig). The *P*-value was calculated with the Wilcoxon test. **C** As an example, predicted secondary structures after (left and green lines in the arc diagram) and before (right and blue lines in the arc diagram) RNA editing of uc002ufm.3 3′ UTR

We used the PARS-seq data of HepG2 cells under normal and ADAR knockdown conditions [10] to assess the changes in the RNA secondary structures in response to RNA editing. Specifically, considering that the PARS-seq data were mainly obtained for the mRNA sequences and that the RNA editing sites are highly enriched in the 3′ UTRs, we focused on the RNA editing sites located in the 3′ UTRs of the coding genes according to the annotation by ANNOVAR [46]. A total of 333 out of 960 A-to-I RNA editing sites, of which at least one RBP showed significant preference toward the edited or unedited RNA, were found in the 3′ UTR of 467 mRNA transcripts. The secondary structures of these 3′ UTR sequences upon editing specific sites were inferred by taking into account the PARS-seq data under normal and ADAR knockdown conditions [47] (Additional file 9: Table S7). As shown in Fig. 5A, alteration of the editing levels due to ADAR knockdown presumably had different consequences on the RNA secondary structures. Interestingly, the 3′ UTR sequences harboring RBP-associated editing sites were much more sensitive to ADAR knockdown than the 3′ UTR sequences harboring the non-RBP-associated editing sites within the RBP-bound RNA regions (Fig. 5B).

For example, the overall structure of the SSB 3′ UTR was changed the most upon ADAR knockdown, which resulted in a more compact RNA structure (Fig. 5C). The local structure where the editing site (chr2: 170668474) resides was also subjected to a dramatic change (Fig. 5C). Therefore, it is a plausible hypothesis that such a change rendered more favorable binding to the RBP DGCR8.

### Correlation between RBP-associated RNA editing events and alternative splicing

A-to-I RNA editing events taking place on pre-mRNA before RNA splicing have been shown to be related to alternative pre-mRNA splicing [5]. On the other hand, many of the RBPs in the present study are known regulators of RNA splicing, e.g., HNRNPC, ILF3, and DROSHA [43, 48–50]. Therefore, we asked whether RBP-associated RNA editing events were related to RNA splicing regulated by RBPs. Here, only the intragenic RNA editing sites falling inside gene bodies were considered.

**Fig. 6:**
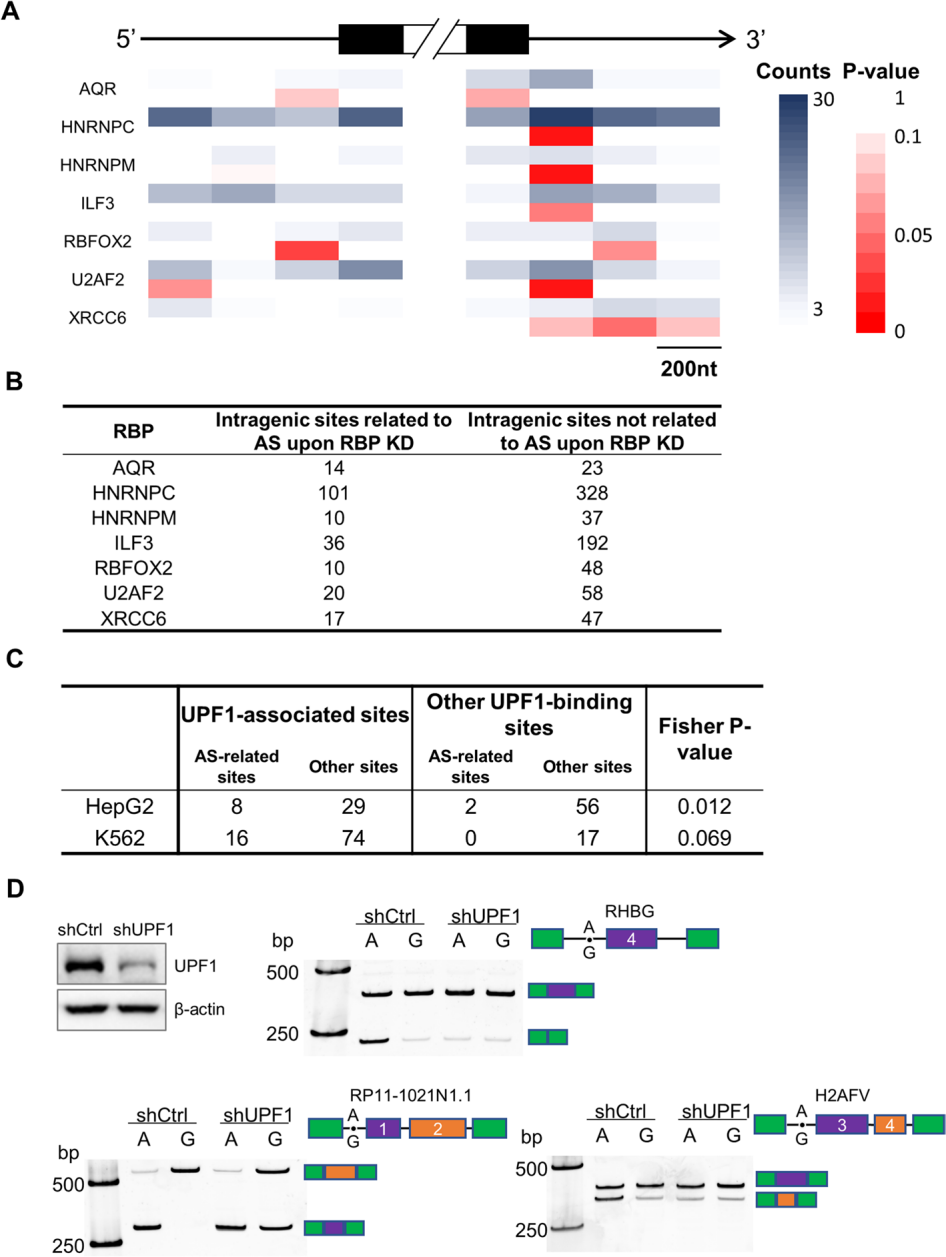
Association between RBP-associated RNA editing events and alternative splicing. **A** Enrichment of the RBP-associated intragenic RNA editing events in K562 near the intron and exon ends. The sequences near the exon and intron ends were binned into 200-nt blocks. The numbers of editing sites located in each block are coded by a blue gradient. *P*-values indicating the significance of the enrichment were estimated by Fisher’s exact test. **B** For the RBP-associated RNA editing sites falling in gene bodies, the sites related to the AS events upon RBP knockdown were counted. See the “Methods” section for the identification of the AS-associated RNA editing sites. **C** Enrichment of UPF1-associated RNA editing sites by AS-related editing events upon ADAR knockdown. The other sites falling in the UPF1 eCLIP peaks but not showing significantly differential editing levels between eCLIP and RNA-seq, i.e., “Other UPF1-binding sites,” were used for comparison by Fisher’s exact test. **D** RNA splicing reporter assays showing patterns of alternative splicing upon editing at an intronic editing site, under the conditions of control or knockdown of UPF1 in HepG2 cells

For some of the RBPs, such as HNRNPC, HNRNPM, ILF3, U2AF2, and XRCC6, their associated intragenic RNA editing sites were found to be enriched toward the 5′ end region of the introns, whereas the editing sites associated with RBFOX2 were enriched near the 3′ end of the introns (Fig. 6A). Furthermore, we obtained alternative splicing (AS) events from the RNA-seq data in ENCODE upon RBP knockdown in HepG2 and K562 cells [24]. Large proportions of the RBP-associated RNA editing sites were located in the AS-related RNA regions upon knockdown of the particular RBP in K562 cells (Fig. 6B), suggesting potential links between the AS events upon RBP perturbation and the preferences of the RBP for specific RNA editing events.

Next, we obtained AS events from the RNA-seq data upon ADAR1 knockdown in HepG2 and K562 cells [24]. The RNA editing sites located in the AS-related RNA regions were then defined as AS-related sites. Interestingly, the UPF1-associated RNA editing sites exhibited the enrichment by these AS-related sites (Fig. 6C). Indeed, a series of splicing reporter assays confirmed alternative pre-mRNA splicing upon editing at particular sites in introns (Fig. 6D, conditions of control). More importantly, these assays further showed that such RNA editing-dependent alternative splicing patterns were either abolished or altered upon knockdown of UPF1 (Fig. 6D). Therefore, these experiments support the insights from the data-mining results above, i.e., the alternative RNA splicing patterns depend on UPF1-associated RNA editing events and the presence of the RBP UPF1.

Indeed, although UPF1 has been best known to function on the 3′ UTR of mRNA transcripts and mediate the NMD process, it has also been shown to colocalize with ADAR1 in supraspliceosomes and in additional nuclear complexes [39], suggesting a link between RNA editing and splicing. Knockdown of UPF1 induced alternative splicing events in zebrafish [51] and human cells [24]. Furthermore, UPF1 has been found to be critical for nonsense-associated altered splicing (NAS) [51–53]. A more recent study further discovered that UPF1 scans pre-mRNAs and directly regulates splicing by interfering with 5′ splice site recognition in *Drosophila* [54]. Therefore, previous reports have established a potent and complicated link between UPF1 and splicing regulation. The observation from our analysis, i.e., the UPF1-associated RNA editing sites are significantly enriched by the AS-related sites, suggests a plausible hypothesis that the editing levels at these sites contribute to splicing regulation by tuning their binding with UPF1. Along this direction, further investigations are needed to elucidate the clear machinery that connects UPF1 binding, RNA editing, and splicing regulation.

## Discussion

The present study focused on the potential association between RNA editing events and the binding of RBPs to their target RNA sequences. Specifically, we performed a comprehensive survey of the binding preferences of 150 RBPs to A-to-I RNA editing events. By taking advantage of the RBP eCLIP-seq data and ultra-deep RNA-seq data in the ENCODE project [23, 55], this survey yielded thousands of RNA editing events showing significantly biased binding preferences by the RBPs in two cell lines, HepG2 and K562.

The preferable binding of RBPs to the edited or unedited RNA could depend on either the RNA sequence or the RNA secondary structure, varying for different RBPs. RNA editing leads to changes of the RNA sequence itself, and it is also well acknowledged that such changes of the RNA sequence usually result in shifts of the RNA secondary structures as well [10, 56, 57]. Therefore, it is quite likely that some RBPs favorably bind to the edited or unedited RNA due to the RBP-binding sequence motifs, while some other RBPs recognize the RNA secondary structure formed by the edited or unedited RNA. For example, both ILF3 and HNRNPC showed strong preferences to RNA editing events (Fig. 3). ILF3 is a well-appreciated dsRNA-binding protein [58]. Interestingly, it has been further shown that ILF3, like ADAR2, binds to certain dsRNA structures with specific sequence features [59]. In other words, both the dsRNA structure and the underlying sequence together determine the binding of ILF3 to the edited RNA. For HNRNPC, its RNA binding depends on HNRNPC recognition motifs such as uridine (U)-rich sequences rather than specific secondary structures [60].

RBPs could have strong preferences for single- or double-stranded RNA. Although ADAR-mediated A-to-I editing is canonically considered a machinery that destabilizes RNA duplexes [56, 57], recent studies have also shown that RNA editing, especially on nonperfect dsRNAs, could further stabilize dsRNA structures [10]. This was confirmed by the analysis of the PARS score changes upon ADAR knockdown, which shows both gain or loss of the double-stranded structures on the editing sites or of the full-length transcripts (Additional file 1: Fig. S11). Therefore, theoretically, the edited RNA sequences could show favorable or unfavorable binding preferences for either dsRNA- or ssRNA-binding proteins.

In addition, despite the various methods for probing RNA secondary structures, it is still challenging to precisely determine the double- or single-stranded status of all regions in large RNA molecules, such as mRNA and lncRNA species. For instance, of all the RNA editing sites included in our analysis, fewer than 2% were probed by PARS-seq, thereby presenting information on their secondary structures. Furthermore, it has been well acknowledged that in vivo RNA structures could be highly dynamic. A multitude of complex factors contribute to defining the structure of an RNA region, such as its binding with RBPs, posttranscriptional processing including RNA editing, the isoform of the RNA transcript, subcellular location, and local conditions (e.g., liquid-liquid phase transitions). Therefore, given that the structural information of most RNA editing sites is unknown, we rely on eCLIP-seq data as a trustworthy survey of the RNA sequences that directly bind with each RBP.

Another major issue of the analysis is that the eCLIP-seq assays were performed with whole cell extracts, whereas the intracellular locations of RBPs are usually unbalanced between the nucleus and the cytosol [24, 61]. Therefore, for an RNA molecule whose editing levels are different in the nucleus and cytosol, a direct comparison between eCLIP-seq and RNA-seq data would introduce systematic errors for estimation of the RBP’s preference toward RNA editing events. This issue has been mostly resolved as discussed below.

First, the intracellular locations of the RNA molecules are highly unbalanced between the nucleus and the cytosol. Ninety percent of the nucleotides are spliced out as introns, and less than 10% of the nucleotides remain in the mature RNA [62]. Furthermore, it is estimated that only approximately 30% of RNA transcripts are processed and exported to the cytoplasm [63]. This has been confirmed by our observation that few of the RNA editing events are detected in both the nuclear and cytosolic fractions (Fig. 1A). Therefore, in general, RNA editing events are specific to the two cellular fractions, which is expected. The RNA editing events carried out by ADAR P110 in the nucleus are mostly in the introns, which are spliced out and kept in the nucleus. On the other hand, the RNA editing events carried out by ADAR P150 in the cytosol should be mostly kept in the cytosol and therefore undetectable in the nucleus. Such nucleus- or cytosol-specific locations of the different RNA species make it feasible to quantitatively compare RBP-associated RNA editing with the overall RNA editing level. Therefore, it is a reasonable assumption that the eCLIP-seq data obtained with whole-cell lysates and the whole-cell RNA-seq data essentially captured the RNA species from the same pool. In addition, whole-cell RNA-seq has the highest sequencing depth, making it more reliable for the assessment of RNA editing profiles.

Furthermore, for the RNA editing sites detected in multiple different cellular fractions (nucleus, cytoplasm, and whole cell), the editing levels of the majority were mostly consistent across different fractions (Fig. 1B). This makes it feasible to compare the RBP eCLIP-seq data with the RNA-seq data without worrying much about the unbalanced RNA editing levels across different cell fractions. Nevertheless, very few RNA editing sites had significantly different editing levels across different fractions (Fig. 1B), and these sites were removed for further analyses.

In summary, given the discussions above, we are convinced that the issue of RBP associated with the different stages of RNA maturation or nuclear/cytosolic location of the RNA should have been largely circumvented. Nevertheless, we used the RNA editing events detected from the RNA-seq data of all three fractions (whole cell, nucleus, and cytosol) as the backgrounds to compare with the RNA editing levels in the RBP eCLIP-seq data. Approximately 50–70% of the RBP-editing associations identified with the cytosolic or nuclear fraction RNA-seq data were also found with the whole-cell RNA-seq data (Additional file 1: Fig. S5).

This study emphasizes the potential impact of RNA editing events on the functions of RBPs that depend on binding to their target RNA sequences. Based on our analyses, the binding preferences for the same RNA editing sites by different RBPs suggest a new type of RBP association network (Fig. 4), which is specifically mediated by RNA editing events. Such a network is different than the previous RBP networks simply based on their binding targets [64, 65], shedding light on a new type of RBP functional association. The information provided by this new RNA editing-based interaction network is echoed by the literature, for example, the coupled functions of HNRNPC and UPF1 in controlling and recycling mis-spiced pre-mRNA [34]. In addition to these previously known interactions, the other novel connections revealed by this network are therefore worth further investigation to elucidate how RNA editing events are involved in mediating the functional connections between different RBPs.

For many of the RNA editing sites, biased binding by RBPs provides potential links between the RNA editing levels and the functions of RBPs, some of which have not been elucidated before, including the regulation of pre-mRNA splicing and high-order RNA structures. It has been well recognized that RNA editing could have a strong impact on both the RNA sequence context and the high-order structure of the RNA [10], which is critical for its binding with many RBPs [14, 45]. This provides plausible mechanisms for at least some of the RBP-associated RNA editing events. Other potential mechanisms include RNA editing facilitated or inhibited by RBP binding and RNA processing, such as splicing, degradation, and translocation.

Many previous studies have reported strong involvement of A-to-I RNA editing events in alternative splicing [5, 66]. Our results highlighted the potential roles of RBPs in connecting RNA editing events and RNA alternative splicing. For example, some of the RBP-associated RNA editing events were significantly enriched toward the 5′ or 3′ ends of the introns (Fig. 6A), and furthermore, many of the RBP-associated RNA editing sites were located in the AS-related regions upon knockdown of the particular RBP (Fig. 6B). Therefore, we believe that these observations provide novel insights into the machinery of the RBP-related regulation of RNA splicing, which could potentially involve the binding of RBPs in favor or disfavor of RNA editing events.

For example, our results provide a potentially plausible hypothesis for the connections between the RBP UPF1, RNA editing, and splicing regulation. We found that UPF1-associated RNA editing sites were highly enriched by AS-related editing events (Fig. 6C), which were revealed by knockdown of ADAR1. AS coupled with NMD (AS-NMD) has been proposed to be an important machinery of gene expression regulation [67, 68]. Our analysis suggests a new hypothesis that UPF1-related RNA splicing regulation could involve biased binding by UPF1 to RNA editing sites. Indeed, preliminary tests with RNA splicing reporter assays confirmed that the AS patterns depend on UPF1-associated RNA editing sites and that such dependency rely on the presence of UPF1 (Fig. 6D).

## Conclusions

In summary, the present study provides a comprehensive survey of RBP-associated RNA editing events, from which new hypotheses and insights could be generated about the connections between RNA editing events and different types of RBP-associated biological processes, such as splicing, translation, and structural shifts.

## Methods

### Data collection and processing

The human genome annotation GRCh37 was downloaded from the UCSC genome browser [69]. The eCLIP-seq data of 150 RBPs and RNA-seq data from different cell fractions (nucleus, cytosol, and whole cell) of HepG2 and K562 were downloaded from the ENCODE project [23, 55]. The RBPs’ binding peaks from the eCLIP-seq data were also obtained from the ENCODE project (bed format). The bam files of eCLIP-seq assays were downloaded from ENCODE for 102 and 120 RBPs in HepG2 and K562, respectively [23, 55]..

The previously annotated A-to-I RNA editing events, which were obtained from the REDIportal [70] database, were used for all the analyses in the current study. The AS events upon ADAR1 knockdown were obtained from a recent large-scale functional survey of the RBPs by ENCODE (ENCSR413YAF) [24].

### Subcellular locations of the RBPs

The current study refers to the experimental investigations of the subcellular locations of 131 RBPs in HepG2 and K562 cell [24]. Intracellular locations of the other remaining 19 RBPs were obtained from GeneCards [61].

### Identification and quantifications of the RNA editing events from the RNA sequencing data

The A-to-I editing events were identified from RNA-seq and eCLIP-seq datasets, by using REDIKnown.py from REDItools v1.0.4 [71] based on the RNA editing database REDIportal [70]. Established by the GTEx project, REDIportal is an archive of the previously annotated RNA editing events in 9642 human RNA-seq samples from 549 individuals [72]. Next, the dbSNP [73] database was used to further remove the apparent RNA editing events that are potentially due to SNPs. Thus, all the RNA editing events from either the RNA-seq or the eCLIP-seq data in this study are previously known and already annotated as RNA editing events.

Specifically, the RNA editing events were identified from RNA-seq or eCLIP-seq data by following a previous instruction [74]: the first six bases of each read were removed; PCR duplicate reads and multiple-mapping reads were removed; only the reads with mapping quality higher than 20 were used, and the bases with quality less than 30 were filtered out. For the RNA editing sites identified above, the total read coverage should be higher than 10, and there should be at least one read supporting the A-to-I editing. As for the eCLIP-seq data, only the RNA editing sites located within the RBP-binding peak were retained for further analyses. Different replicates of the RNA-seq data were merged for identification and quantifications of the RNA editing sites. The editing levels were calculated by dividing the reads with A-to-I conversion by the total read depth at the editing site.

### Overall assessment of the RBP-associated RNA editing sites

For each RBP, the editing levels of the RNA editing sites located within the RBP-binding peaks were summarized and compared to the editing levels of the same group of editing sites in the RNA-seq data. The Wilcoxon signed-rank test was used to assess the overall difference between the two distributions. The differences with *P*-value lower than 1×10^−5^ were deemed significant.

### Assessment of the RBP’s binding preference to specific RNA editing sites

For a particular RBP and each specific RNA editing site falling in the RBP-binding peaks, the Fisher exact test [75] was introduced to assess the differential editing levels in the eCLIP-seq data and in the RNA-seq data. *P*-value was then used as a statistical assessment of the differential editing in the RBP-bound RNA editing sites. Finally, the editing sites with the differential editing levels higher than 10% and the *P*-value lower than 0.05 were deemed as the RBP favored (editing level higher in the eCLIP-seq data) or disfavored (editing level higher in the RNA-seq data) editing sites.

### Inference of the RNA secondary structures with PARS-seq data

The PARS-seq data of HepG2 cells was downloaded from GEO (GSE100210). The reads were aligned to human 3′ UTR sequences by bowtie v1.2.3 [76]. As previously instructed [47], such information was then used as constraints for inference of the secondary structures of the 3′ UTRs using the algorithm RNA-fold (v2.4.14) with default parameters [77]. R4RNA v1.14.0 [78] was used to draw the arc diagrams showing the base-pairing schemes of the RNA sequences.

### Identification of the RNA editing sites related to the alternative splicing events

Differential alternative splicing (AS) events upon knockdown of each RBP or ADAR1 were downloaded from the ENCODE project (ENCSR413YAF) [24]. Specifically, rMATS v3.2.1 beta was used to identify the differential AS events from the RNA-seq data [79]. The AS events with *P*-values lower than 0.05 were categorized into 5 types, i.e., SE (skipped exon), MXE (mutually exclusive exons), A3SS (alternative 3′ splicing site), A5SS (alternative 5′ splicing site), and RI (retained intron). For each of these 5 AS types, the RNA editing sites located within the event coordinates (reported by rMATS in the “Events specific columns”) were counted as the AS-related sites.

### Protein expression and purification

Two RNA-binding domains of human ILF3 (ILF3 dsRBD, residues 398-590) and hnRNPC (hnRNPC dsRBD, residues 16-57) were used for electrophoretic mobility shift assay (EMSA). ILF3 dsRBD was cloned into pGood6p vector with N-GST tag, and hnRNPC dsRBD was cloned into pSUMOH10 vector with 10*His tag. These two constructs were expressed in *Escherichia coli* and induced overnight with 1 mM isopropyl--D-1 thiogalactopyranoside (IPTG) at 18°C. Cells were lysed in 25 mM Tris HCl pH 8.0, 150 mM NaCl, and 10 mM imidazole. The proteins were purified with GSH or Ni^2+^-NTA resin (GE Healthcare) at 4°C and eluted in 25 mM Tris HCl pH 8.0, 150 mM NaCl, 300 mM imidazole. The proteins were dialyzed into 25 mM Tris HCl pH 8.0, 150 mM NaCl overnight with 1 mg TEV protease to remove the tags. TEV protease and GST were then removed from the proteins using heparin sepharose or mono S cation exchange chromatography and eluted with a salt gradient to 1 M NaCl. The proteins were further purified by size exclusion chromatography using a Superdex S75 column (GE Healthcare).

### Electrophoretic mobility shift assays

For EMSA assays, synthesized RNA labelled with a 5′fluorescent DY781 dye and containing a three base extension was used (5′-DY781-AGUGGCUUAUGCCUGUA/GAUCCCAACAC). Unlabeled RNA was used for competition assays.

Protein-RNA-binding reactions, containing 1 μM labelled RNA and 3 μM ILF3 or hnRNPC in a 10 μl reaction volume, were carried out on ice for 45 min. Competition assays were also carried out in a 10-μl reaction volume. A 1:1 molar ratio complex of RNA and proteins was prepared by incubation on ice for 1 h. The complex at a final concentration of 0.5 μM was mixed with 50 μM unlabeled competitor RNA. Monoclonal antibodies specific for ILF3 (Abcam, ab92355) and hnRNPC (Abcam, ab133607) were added to the binding reaction after complex formation. A 6% polyacrylamide gel in 0.5× Tris/Borate/EDTA buffer (TBE) was pre-run at 4°C for 1 h. Samples were then mixed with 2 μl of native gel loading buffer to a total volume of 6 μl and run on the gel for 2 h. The gel was scanned on a LICOR Odyssey fluorescent infrared scanner at 800 nm. Images were converted to greyscale by ImageJ.

### Cell culture and transfection

HEK293FT cells and HepG2 cells were cultured with DMEM high glucose with 10% fetal bovine serum (FBS). Forward transfection was carried out with lipofectamine 2000 (Invitrogen) according to the manufacturer’s instructions.

### Lentivirus production and construction of stable cell lines

The shRNA plasmids were obtained from Sigma-Aldrich (TRCN0000022258 for UPF1 and TRCN0000050849 for negative control). For packaging of lentivirus, 70% confluent HEK293FT cells in Flask T-175 were transfected with 10 μg of the shRNA vector with 15 μg and 10 μg of Δ8.9 envelope and Vsv-G packaging vectors, respectively. Six hours after transfection, the medium was replaced with a fresh medium. Infectious lentivirus supernatant was harvested at 72 h post-transfection. After centrifugation, lentivirus supernatant was filtered through 0.45-μm PVDF filters (Millex, SLHV033RB), aliquoted, and stored at −80°C. The lentivirus-containing supernatant supplemented with 8 μg/mL of polybrene (Sigma-Aldrich, H9268) was used to infect the HepG2 cells. Twenty-four hours after infection, the HepG2 cells were selected in a medium containing 2 μg/mL puromycin (Amresco, J593) and passaged under continuous selection for at least 2 passages before the experiments.

### Splicing reporter assays

Reporter plasmid pZW4 was a kind gift from Zefeng Wang’s lab at the Partner Institute for Computational Biology Chinese Academy of Sciences and Max Planck Society, Shanghai. In this reporter construct, the cDNA sequence of GFP was divided into 2 exons. Arranged between these two exons were the exons and introns being tested for alternative splicing such as exon skipping. Site-directed mutagenesis was carried out to induce A to G mutations at particular intronic sites. All constructs were sequenced to confirmed correct insert before transfection.

Total RNA was isolated and purified using TRIzol (Invitrogen) followed by DNase I treatment. The reverse transcription was carried out with High-Capacity cDNA RT Kit with random primers (Invitrogen, 4368814). The PCR primers used in this assay are GFP-F (AGTGCTTCAGCCGCTACCC) and GFP-R (GTTGTACTCCAGCTTGTGCC).

## Supplementary Information

Additional file 1: Fig. S1. Statistics of the RNA editing events. Fig. S2. Cell specificity of the A-to-I RNA editing events in HepG2 and K562. Fig. S3. Statistics of the RNA editing sites falling in the RBP-binding regions. Fig. S4. Distributions of the editing levels in RBP eCLIP and RNA-seq data. Fig. S5. Overlaps between the RBP-associated editing sites identified by comparing RBP eCLIP and RNA-seq of 3 cellular fractions. Fig. S6. Numbers of the RBP-associated RNA editing events. Fig. 7. Summary of the RBP-associated RNA editing events in the same RBP-binding regions. Fig. S8. RBP interaction networks reconstructed with two methods. Fig. S9. The RBP interaction network based on the RBP-associated editing sites, highlighting different RNA processes. Fig. S10. The RBP interaction network based on the overlapping eCLIP peaks, highlighting different RNA processes. Fig. S11. RNA secondary structure differences before and after ADAR knockdown in HepG2.

Additional file 2: Table S1. Intracellular locations of RBPs.

Additional file 3: Table S2. Summary of RNA editing sites detected in each fraction of K562 and HepG2.

Additional file 4: Table S3. RNA editing sites covered in RBP eCLIP data.

Additional file 5: Table S4. Comparison of the overall editing levels between RBP eCLIP and RNA-seq data in K562 and HepG2.

Additional file 6: Distributions of RNA editing levels in RBP eCLIP and RNA-seq data.

Additional file 7: Table S5. Annotations of the statistically significant RBP-associated RNA editing sites in K562 and HepG2.

Additional file 8: Table S6. Survival-related RNA editing sites in cancers, data obtained from Han, L., et al., Cancer Cell, 2015.

Additional file 9: Table S7. Statistics of 3’ UTR energy changes in HepG2 upon ADAR knockdown.

Additional file 10. Uncropped images for the blots in figures 3, 4 and 6.

Additional file 11. Review history.

## Data Availability

The RBPed pipeline has been deposited in github (https://github.com/YangLabProject/RBPed) [80] and Zenodo (10.5281/zenodo.6838048) [81]. The source code is released under MIT License (http://opensource.org/licenses). The eCLIP-seq data of 150 RBPs and RNA-seq data of HepG2 and K562 were downloaded from the ENCODE project (ENCSR456FVU) [23, 82]. The AS events upon ADAR1 knockdown were obtained from a recent large-scale functional survey of the RBPs by ENCODE (ENCSR413YAF) [24, 83]. The PARS-seq data of HepG2 cells was downloaded from GEO (GSE100210) [84].
