# Supplementary material for: Survey of the binding preferences of RNA-binding proteins to RNA editing events
Source: Genome Biol. 2022 Aug 4;23:169. doi: 10.1186/s13059-022-02741-8 (PMC9351184; doi:10.1186/s13059-022-02741-8)
Supplement: Supplementary file 1 — Additional file 1: Fig. S1. Statistics of the RNA editing events. Fig. S2. Cell specificity of the A-to-I RNA editing events in HepG2 and K562. Fig. S3. Statistics of the RNA editing sites falling in the RBP-binding regions. Fig. S4. Distributions of the editing levels in RBP eCLIP and RNA-seq data. Fig. S5. Overlaps between the RBP-associated editing sites identified by comparing RBP eCLIP and RNA-seq of 3 cellular fractions. Fig. S6. Numbers of the RBP-associated RNA editing events. Fig. 7. Summary of the RBP-associated RNA editing events in the same RBP-binding regions. Fig. S8. RBP interaction networks reconstructed with two methods. Fig. S9. The RBP interaction network based on the RBP-associated editing sites, highlighting different RNA processes. Fig. S10. The RBP interaction network based on the overlapping eCLIP peaks, highlighting different RNA processes. Fig. S11. RNA secondary structure differences before and after ADAR knockdown in HepG2. [file 13059_2022_2741_MOESM1_ESM.pdf]

Supplementary Figures

Survey of the binding preferences of RNA-binding proteins to RNA editing events

Xiaolin Hu[1,3,4,*], Qin Zou[2,3,4,*], Li Yao[3,4], Xuerui Yang[3,4,#]

1 School of Public Health, Shanghai Jiao Tong University School of Medicine, Shanghai 200025, China

2 Key Laboratory of Molecular Medicine and Biotherapy, School of Life Science, Beijing Institute of Technology, Beijing 100081, China

3 MOE Key Laboratory of Bioinformatics, School of Life Sciences, Tsinghua University, Beijing 100084, China

4 Center for Synthetic & Systems Biology, Tsinghua University, Beijing 100084, China

* Co-first authors

# Correspondence: Xuerui Yang, School of Life Sciences, Tsinghua University, Beijing 100084, China. Tel: 86-10-62783943. Email: yangxuerui@tsinghua.edu.cn

Fig. S1

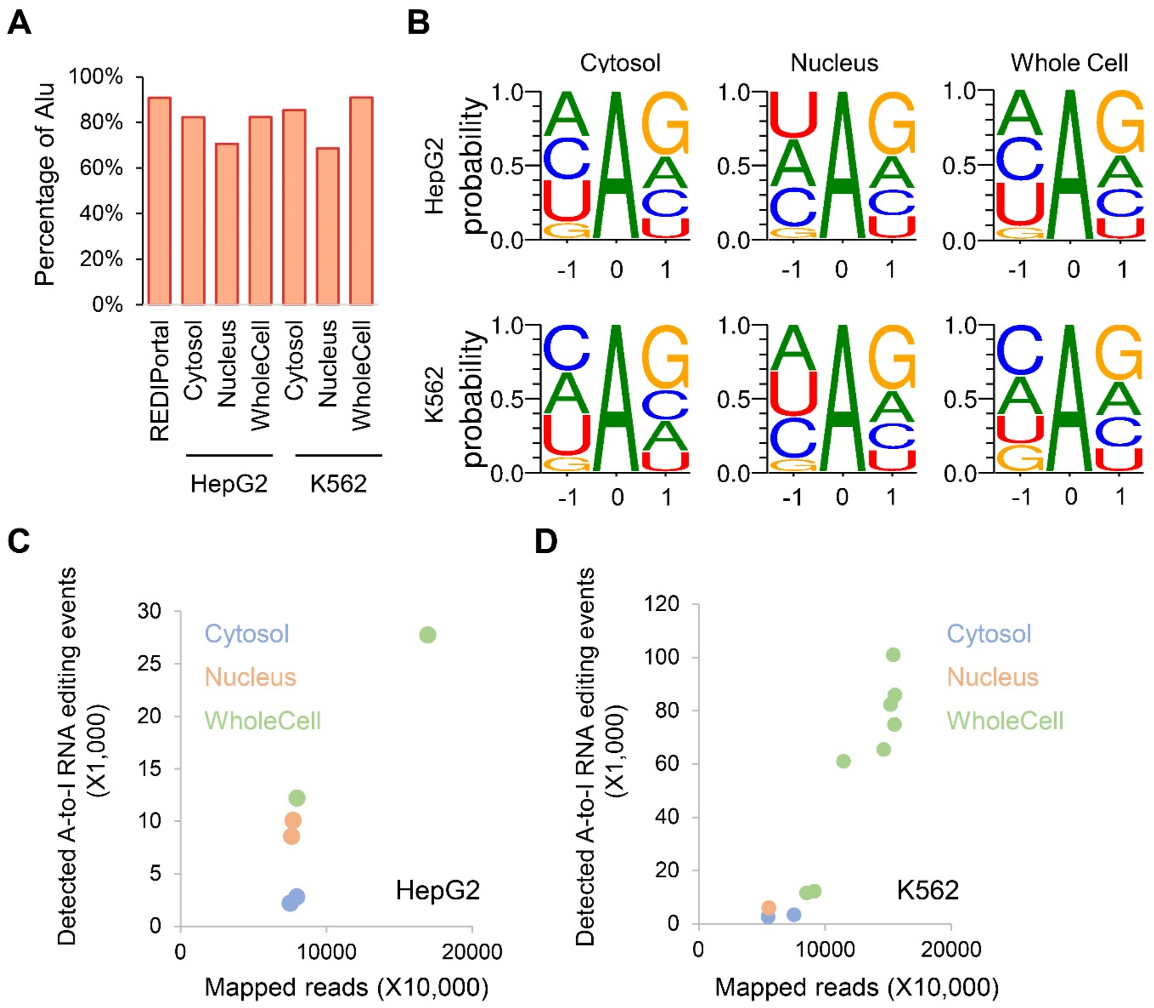

Fig. S1. Statistics of the RNA editing events.

A. Proportions of the RNA editing sites in the present study and in the REDIPortal data base that are found in the Alu elements.

B. Sequence motifs around the RNA editing sites in the present study.

C, D. Numbers of RNA editing events detected from the RNA-seq data in K562 (C) and HepG2 (D). Each dot represents the data of one replicate.

Fig. S2

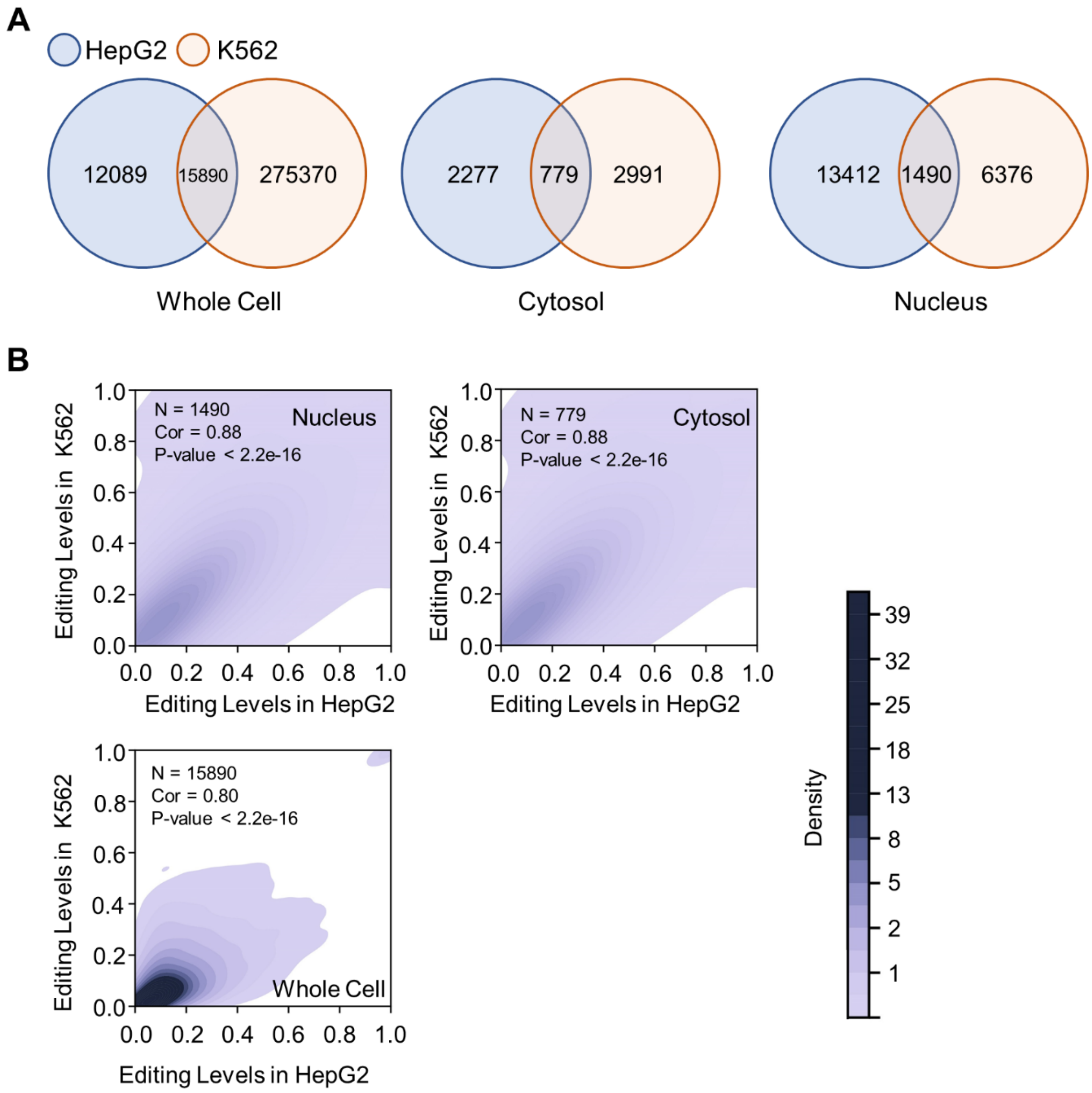

Fig. S2. Cell specificity of the A-to-I RNA editing events in HepG2 and K562.

A. Overlaps between the RNA editing events detected in HepG2 and K562.

B. Pearson correlations of the RNA editing levels between HepG2 (X-axis) and K562 (Y-axis). Each dot represents an editing site, and the total number in each plot is provided (N). Dot density is colored with blue gradient.

Fig. S3

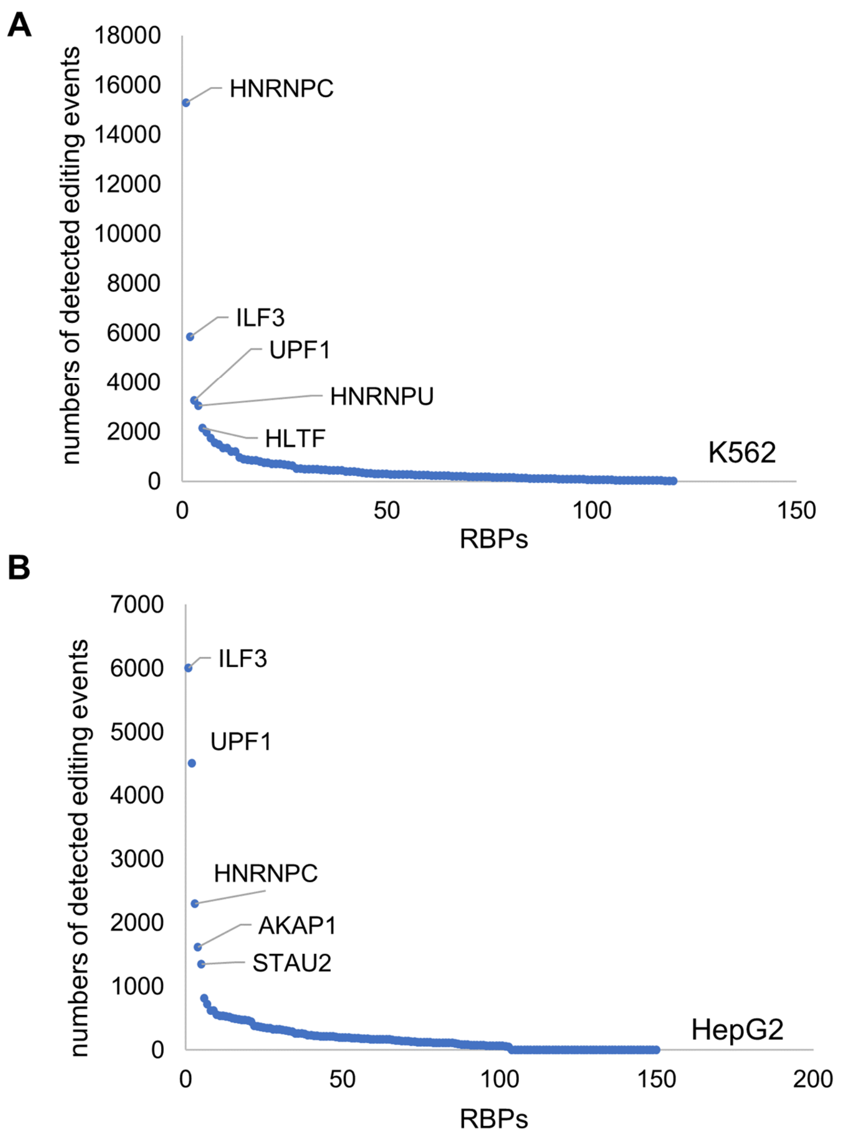

Fig. S3. Statistics of the RNA editing sites falling in the RBP-binding regions.

Numbers of the A-to-I RNA editing events detected in the eCLIP peaks of each RBP in K562 (A) and HepG2 (B).

Fig. S4

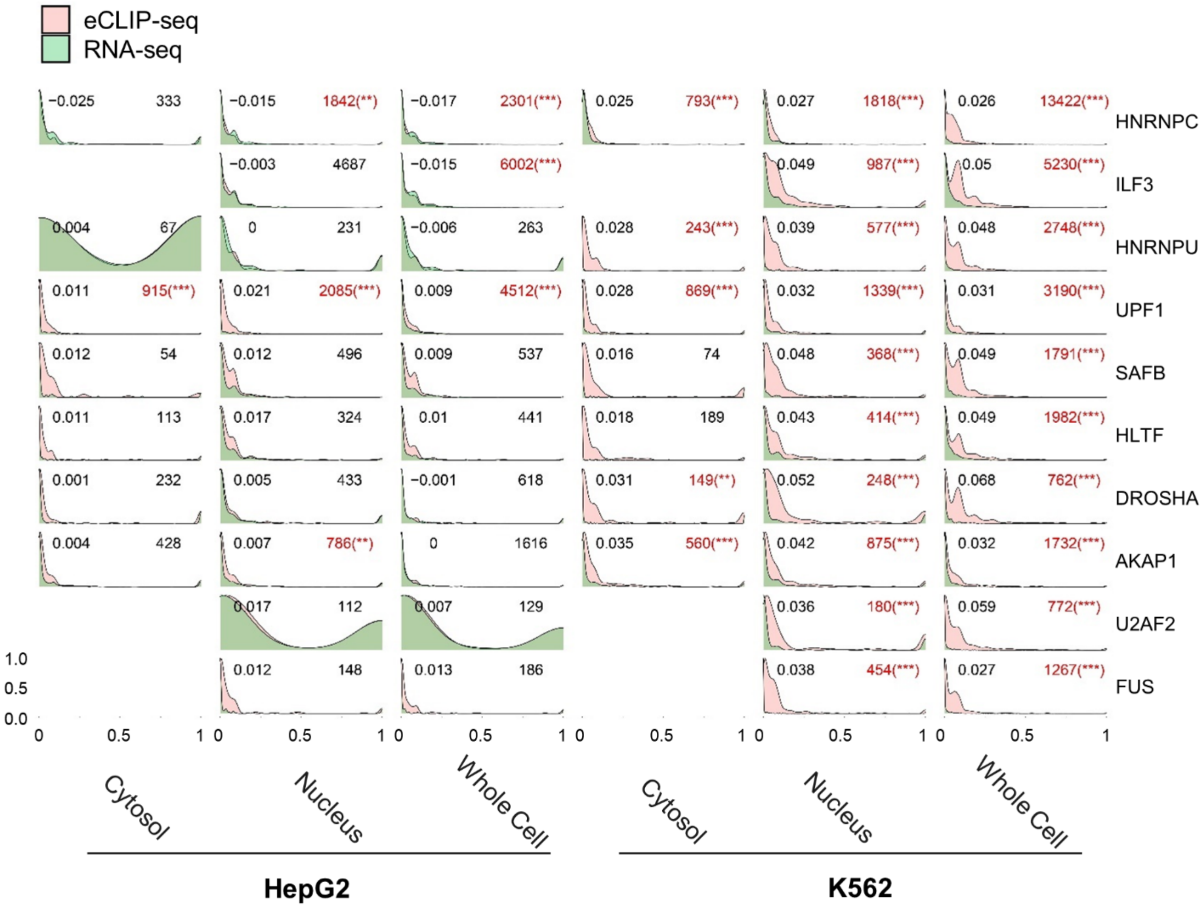

Fig. S4. Distributions of the editing levels in RBP eCLIP and RNA-seq data.

Distributions of the editing levels of the editing sites in each RBP eCLIP and the RNA-seq data. Numbers of A-to-I RNA editing events detected in both the RBP eCLIP and RNA-seq data are provided to the top right corner of each subplot. P-values were calculated by paired Wilcoxon signed test. The significant tests, with a cutoff of 10^{-5}, are marked in red. **: P-value less than 10^{-5}; ***: less than 10^{-10}. The median differential editing level of all the editing sites in each subplot is provided in the top left corner.

Fig. S5

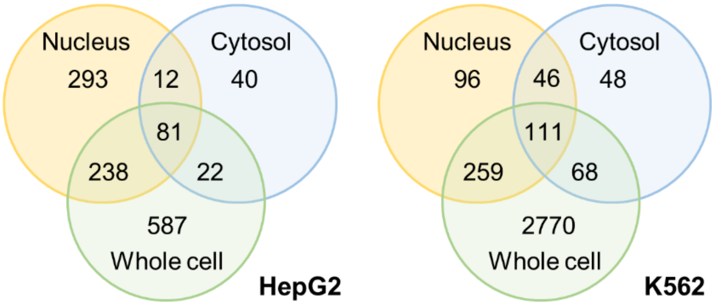

Fig. S5. Overlaps between the RBP-associated editing sites identified by comparing RBP eCLIP and RNA-seq of 3 cellular fractions.

For each RBP, the RBP-associated RNA editing sites were identified by comparing the RBP eCLIP data with RNA-seq of the nucleus, cytosol, and whole cell lysates of HepG2 and K562. Overlaps between the results of the 3 comparisons above were counted and summarized.

Fig. S6

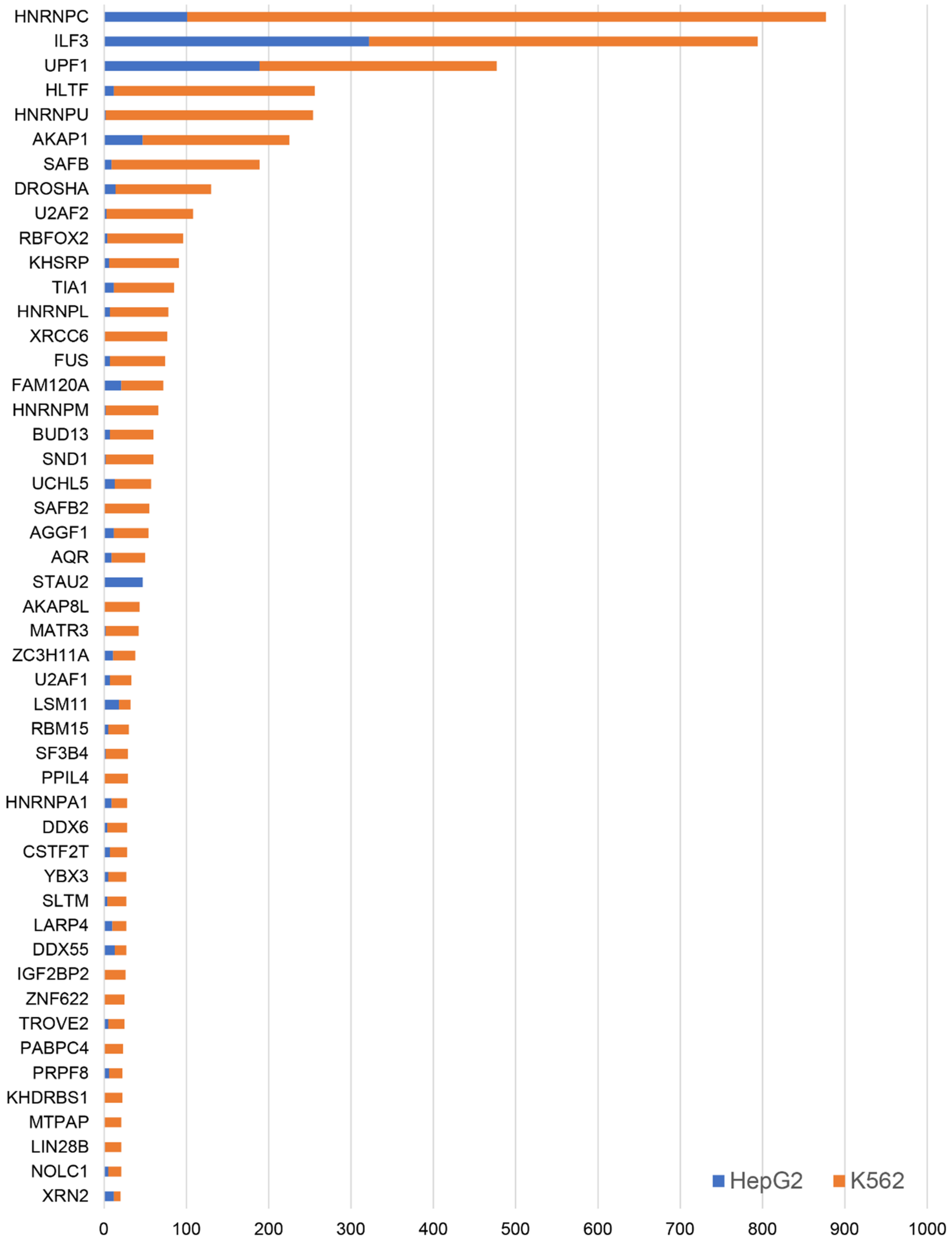

Fig. S6. Numbers of the RBP-associated RNA editing events

Numbers of the RBP-associated RNA editing events with statistical significance in K562 and HepG2.

Fig. S7

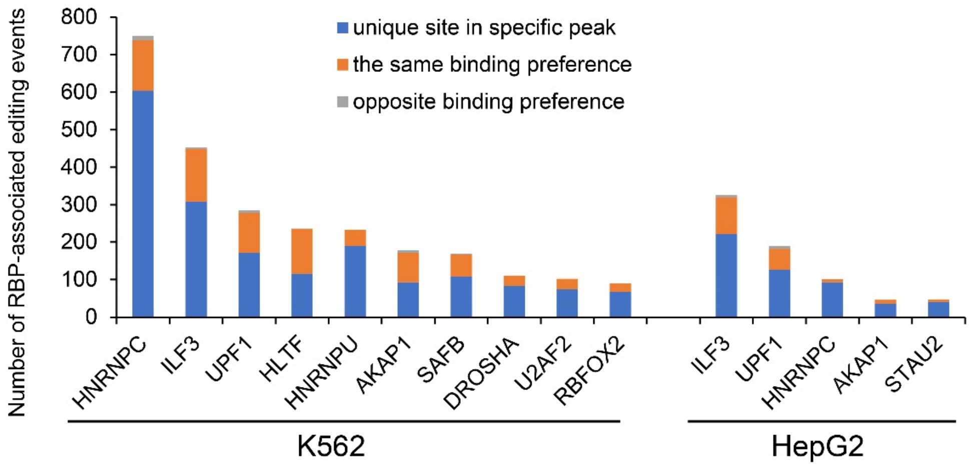

Fig. 7. Summary of the RBP-associated RNA editing events in the same RBP-binding regions.

For each RBP, the target RNA regions harboring the editing sites that are significantly associated to the RBP were divided into 3 groups: regions with just one editing site, regions with multiple sites showing the same direction of binding preferences to the RBP, and the regions with multiple sites with opposite binding preferences to the RBP.

Fig. S8

A

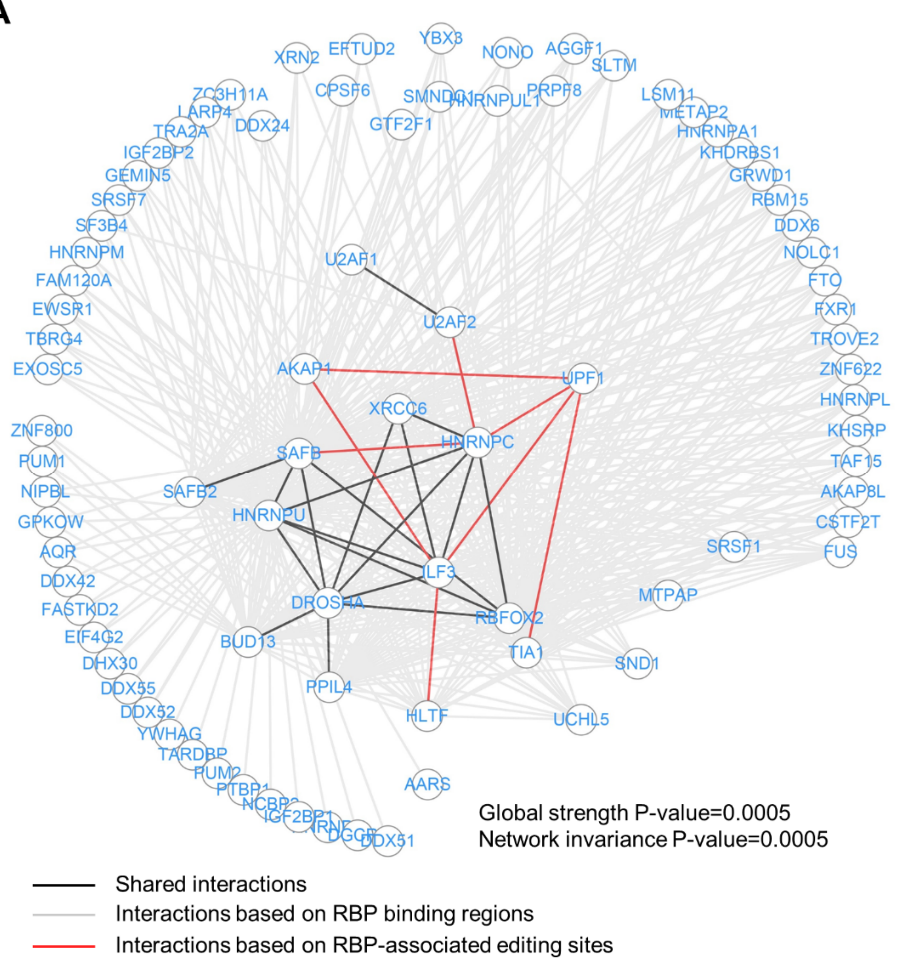

XRN2
EFTUD2
YBX3
NONO
AGGF1
SLTM
ZC3H11A
LARP4
CPSF6
HNRNPUL1
PRPF8
LSM11
METAP2
TRA2A
DDX24
GTF2F1
HNRNPA1
IGF2BP2
KHDRBS1
GEMIN5
GRWD1
SRSF7
RBM15
SF3B4
DDX6
HNRNPM
U2AF1
NOLC1
FAM120A
FTO
EWSR1
U2AF2
FXR1
TBRG4
TROVE2
EXOSC5
AKAP1
ZNF622
UPF1
HNRNPL
XRCC6
ZNF800
HNRNPC
KHSRP
PUM1
SAFB
TAF15
NIPBL
SAFB2
AKAP8L
GPKOW
HNRNPU
CSTF2T
SRSF1
AQR
FUS
ILF3
DDX42
MTPAP
FASTKD2
DROSHA
RBFOX2
EIF4G2
BUD13
TIA1
DHX30
SND1
DDX55
PPIL4
DDX52
HLTF
UCHL5
YWHAG
TARDBP
PUM2
AARS
PTBP1
DDX51
Global strength P-value=0.0005
Network invariance P-value=0.0005
Shared interactions
Interactions based on RBP binding regions
Interactions based on RBP-associated editing sites

B

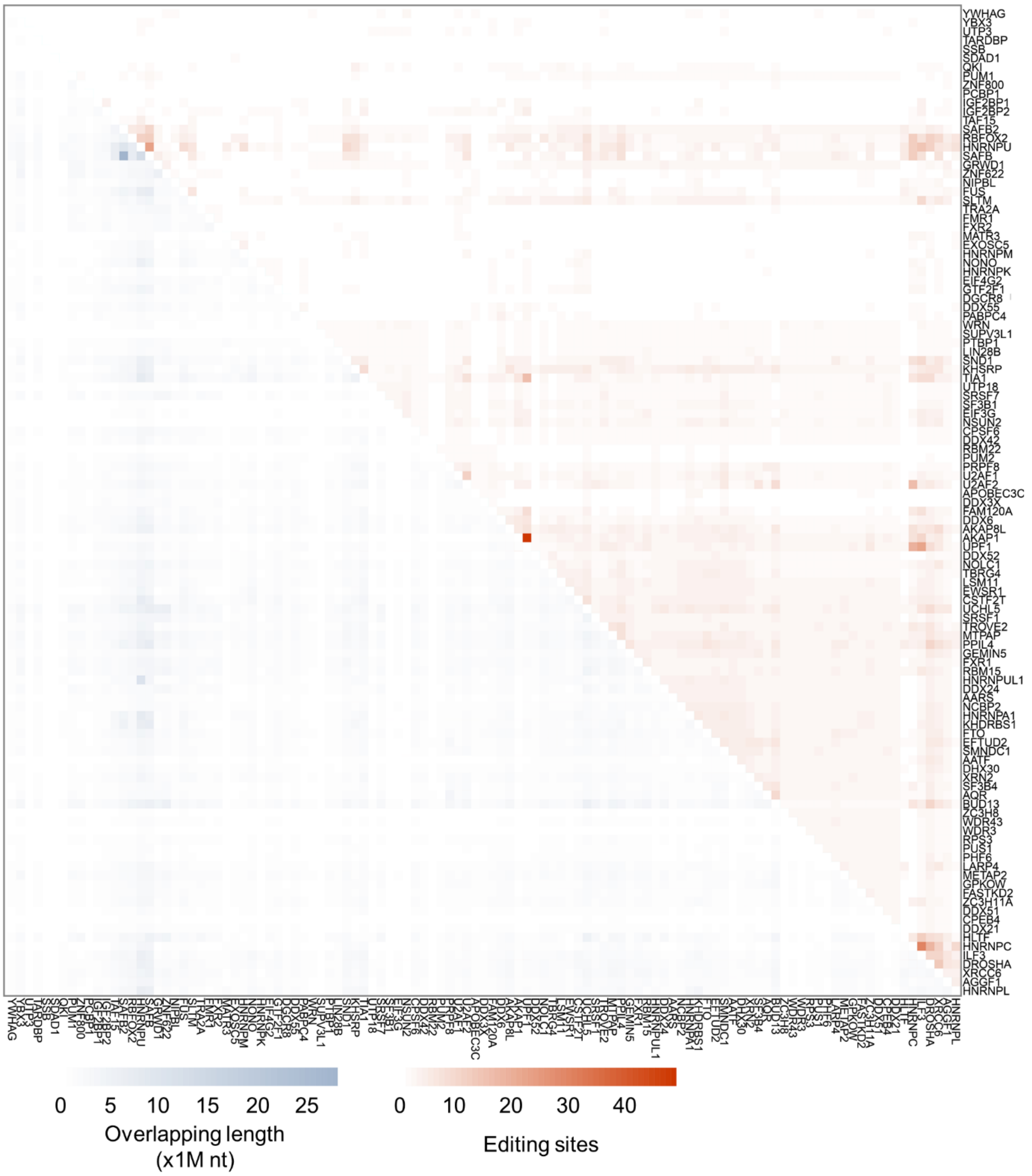

Fig. S8. RBP interaction networks reconstructed with two methods.

A. Interactions between RBPs were inferred based on their overlapping binding regions from eCLIP data (grey) or based on the RBP-associated RNA editing sites in common (red). The interactions inferred by both methods are shown as black edges.
B. Overlaps between the RBP-binding regions (lower left) or between the RBP-associated RNA editing sites (Top right), for each pair of the RBPs.

Fig. S9

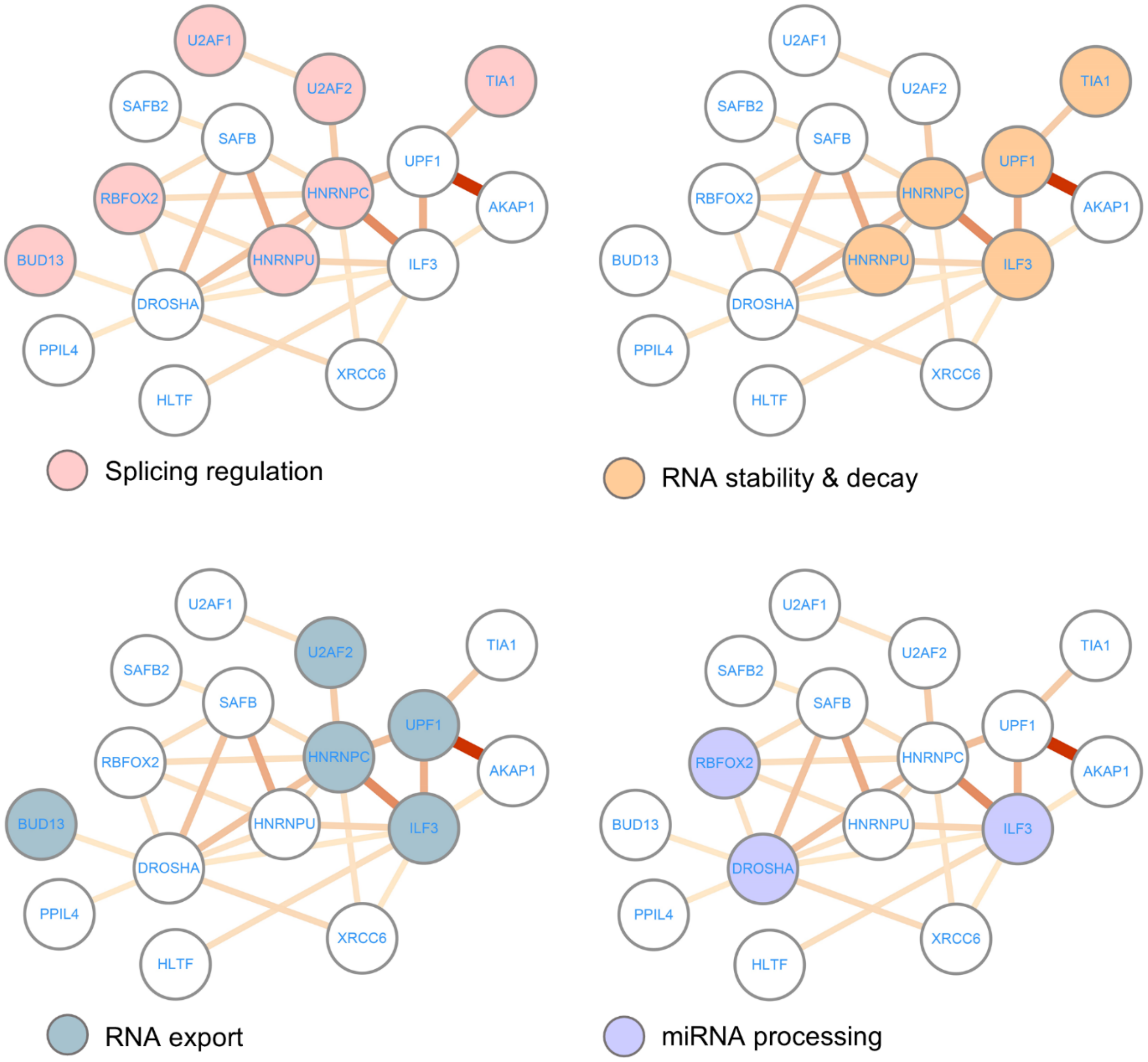

Fig. S9. The RBP interaction network based on the RBP-associated editing sites, highlighting different RNA processes.

RBP interaction network showing the overlapping RNA editing events associated to each pair of the RBPs in K562 (also shown in Fig. 4B). The RBPs involved in different RNA-related processes are highlighted in different colors.

Fig. S10

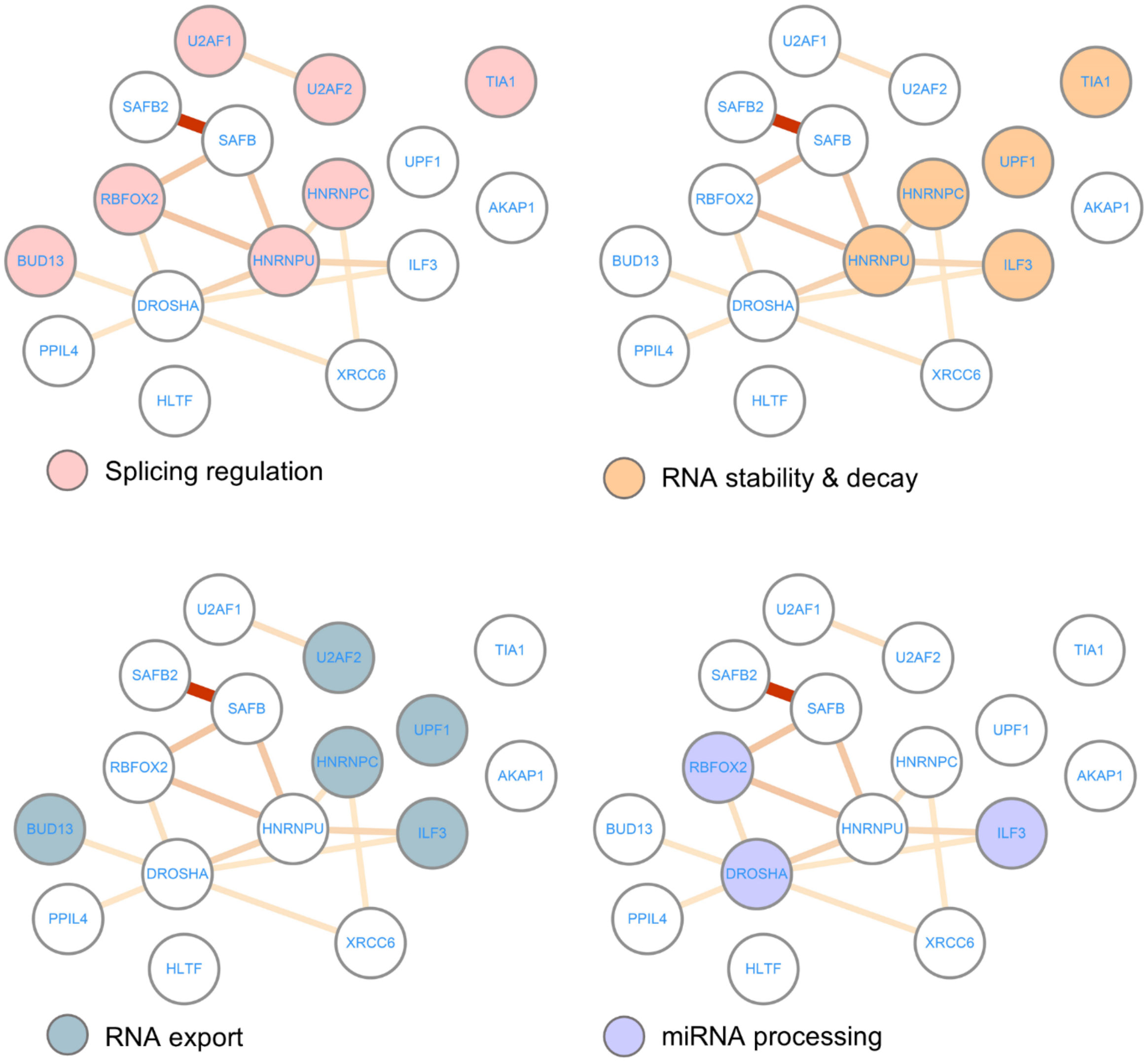

Fig. S10. The RBP interaction network based on the overlapping eCLIP peaks, highlighting different RNA processes.

RBP interaction network based on the overlapping RBP-binding RNA regions (also shown in Fig. 4C). The RBPs involved in different RNA-related processes are highlighted in different colors.

Fig. S11

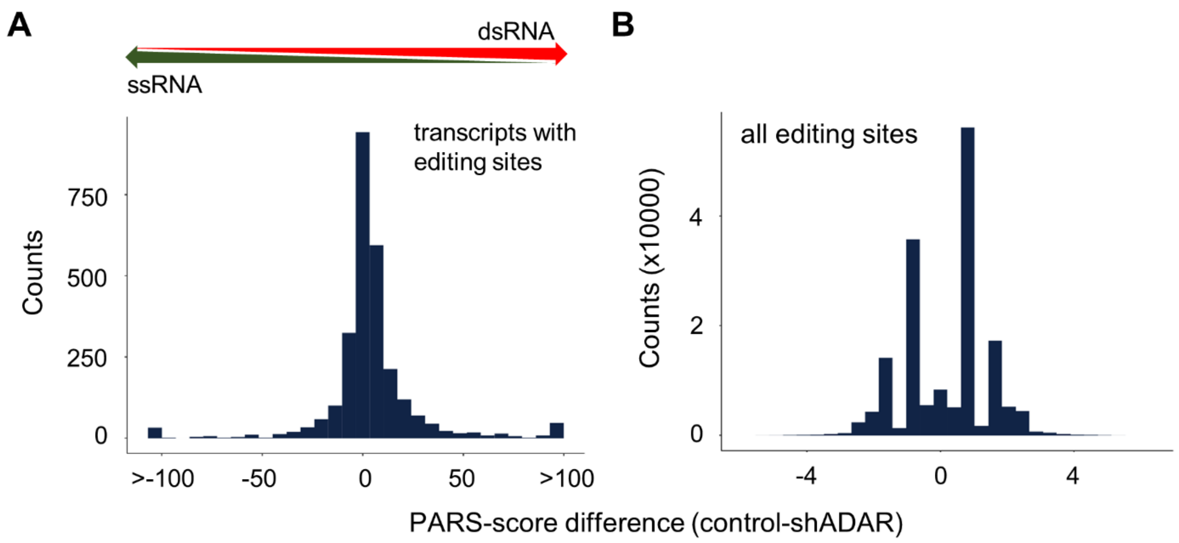

Fig. S11. RNA secondary structure differences before and after ADAR knockdown in HepG2.

PARS-score changes of the transcripts with RNA editing sites (A) and of the specific RNA-editing sites (B) were calculated by comparing the control cells against the shADAR cells. PARS-score was defined as log2(reads of V1 divided by reads of S1), in which RNase V1 digests dsRNA and RNase S1 digests ssRNA. Therefore, the PARS-score differences above 0 indicate higher probabilities of the dsRNA structures, whereas the differences below 0 indicate more ssRNA conformations.
