# Supplementary figures and images for: Survey of the binding preferences of RNA-binding proteins to RNA editing events
Source: Genome Biol. 2022 Aug 4;23:169. doi: 10.1186/s13059-022-02741-8 (PMC9351184; doi:10.1186/s13059-022-02741-8)

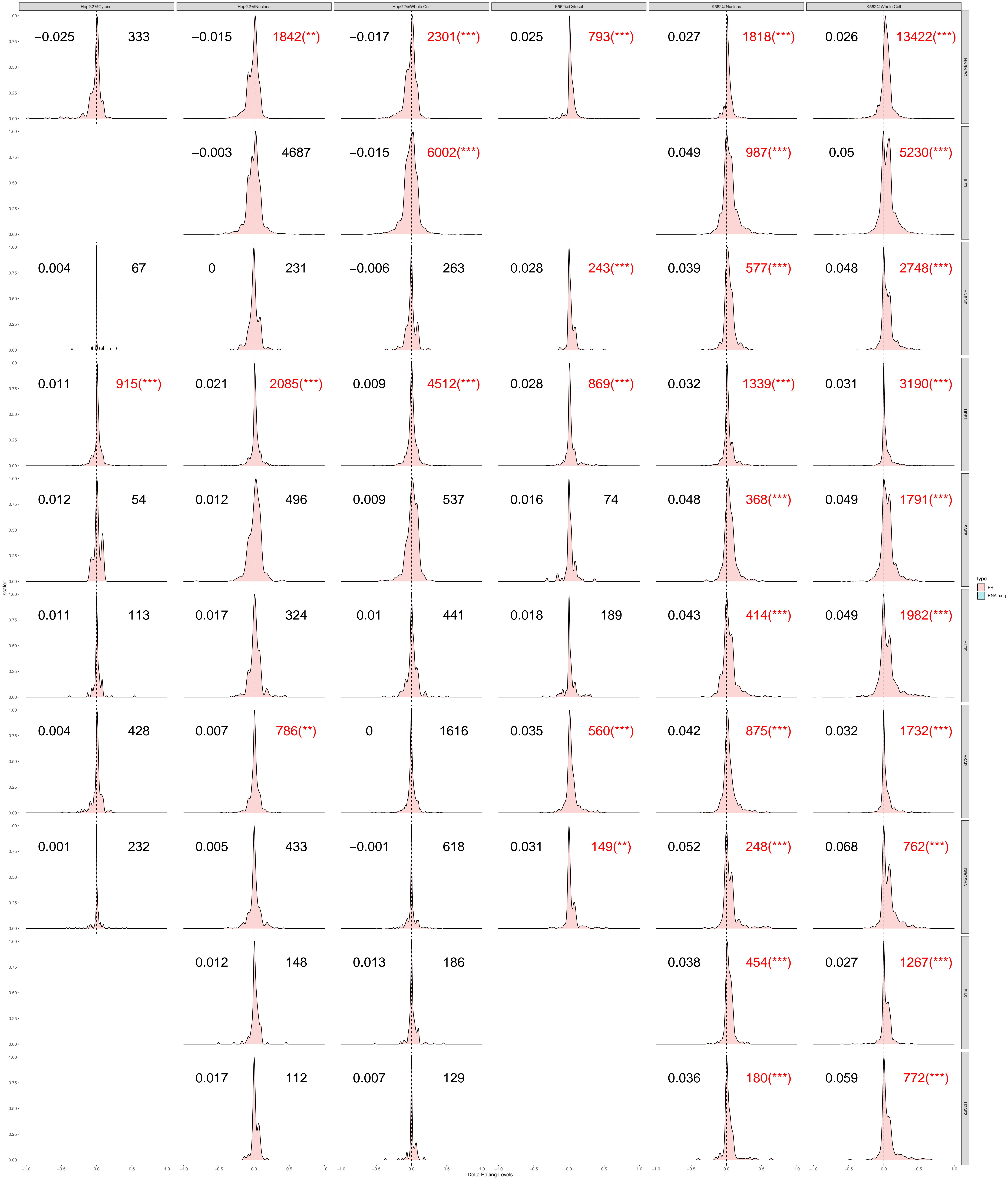

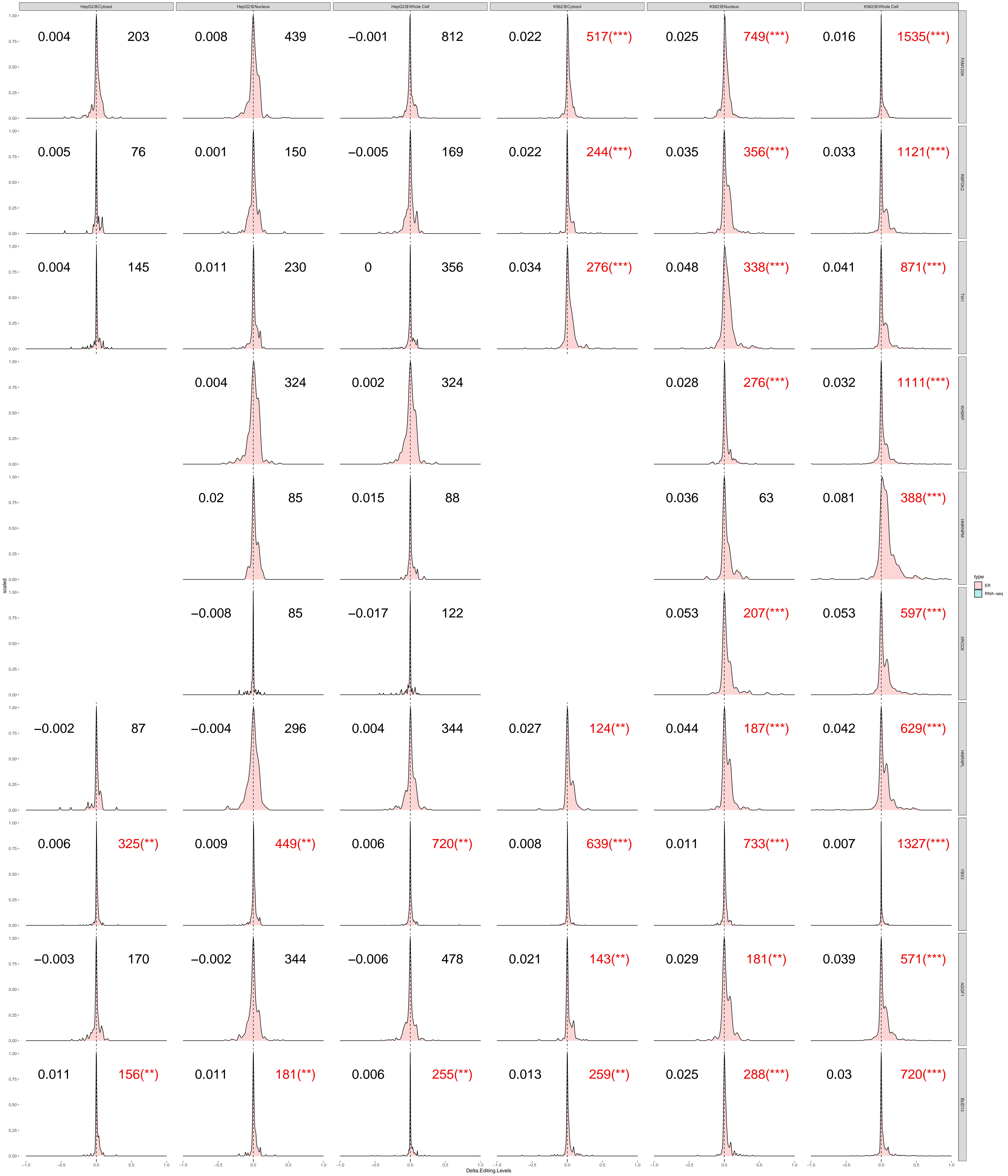

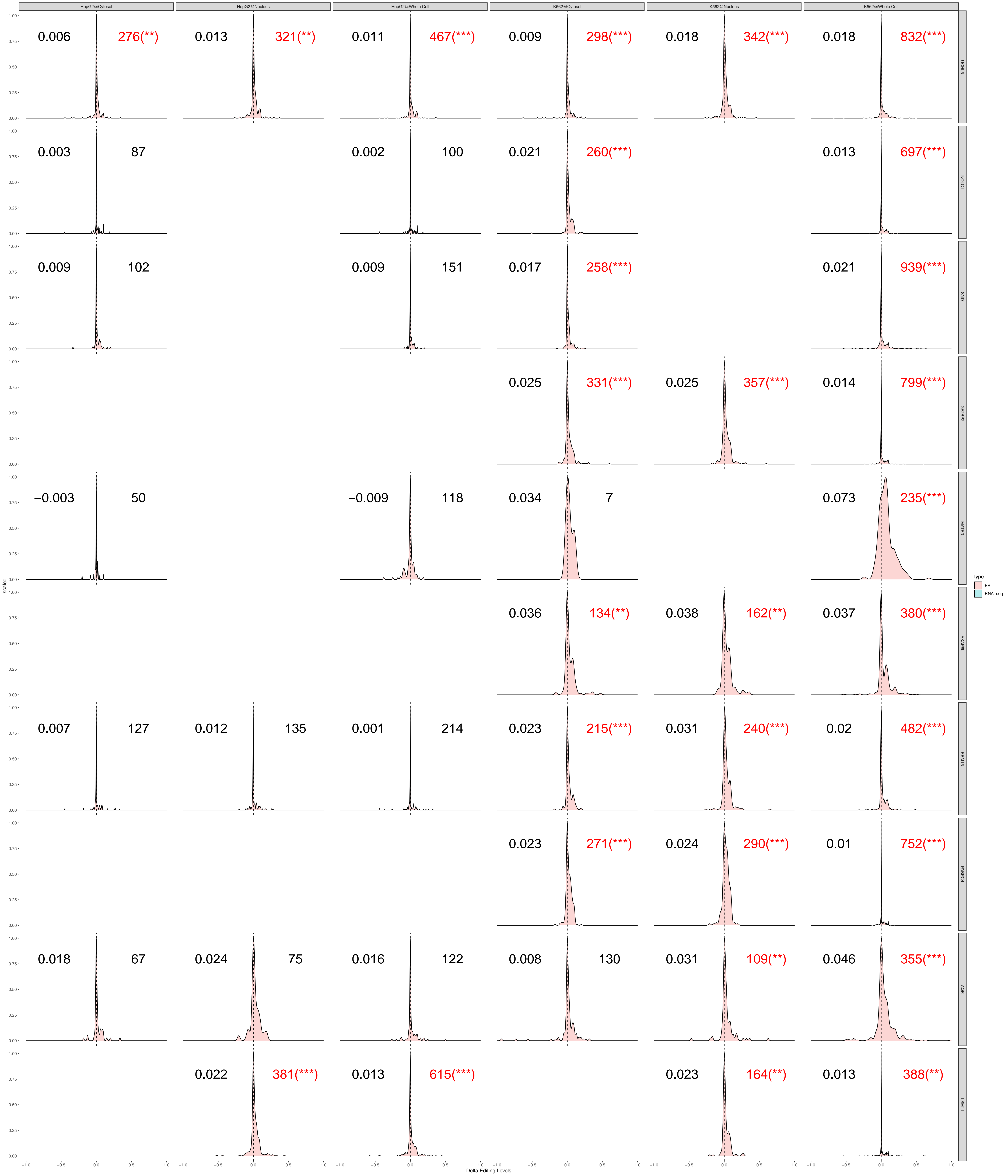

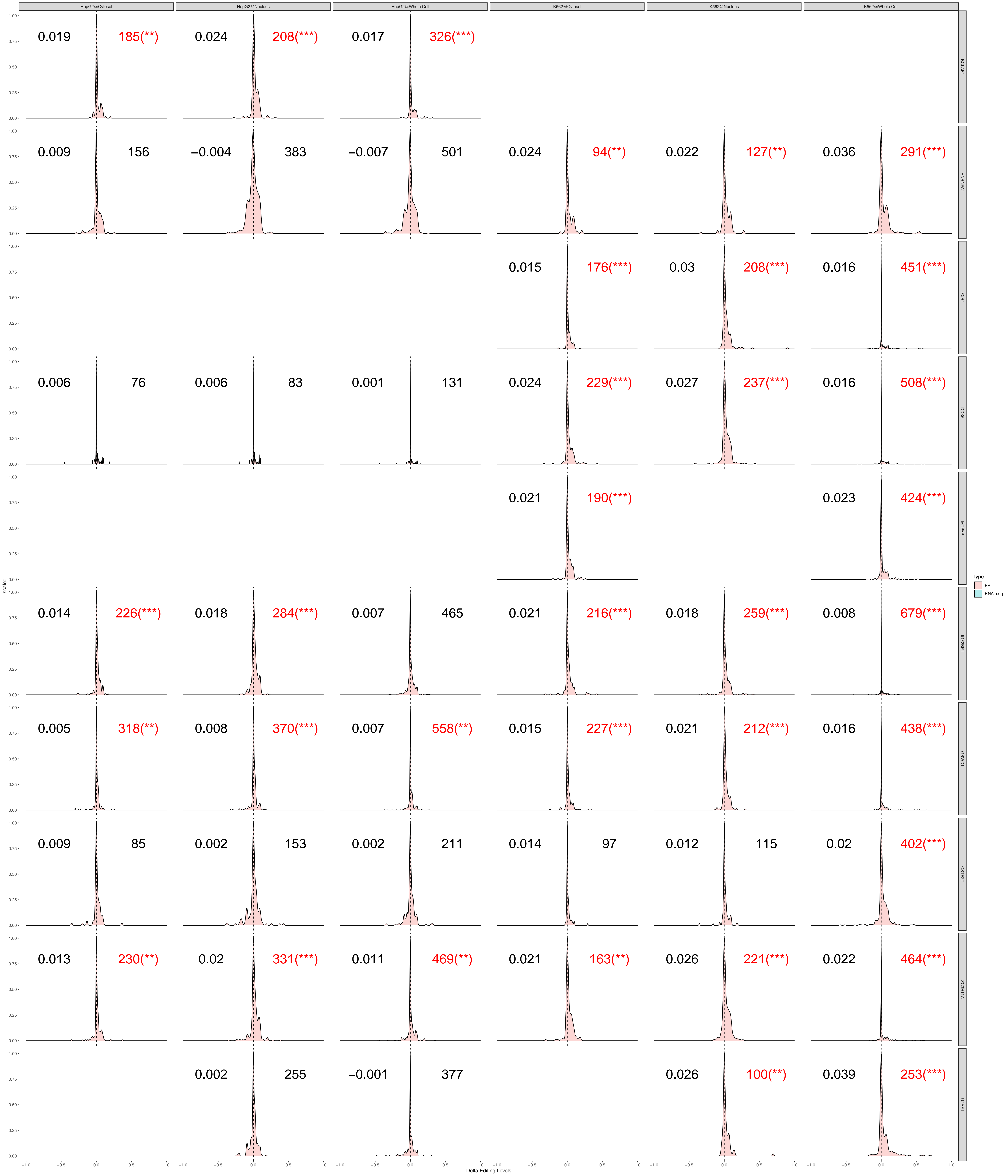

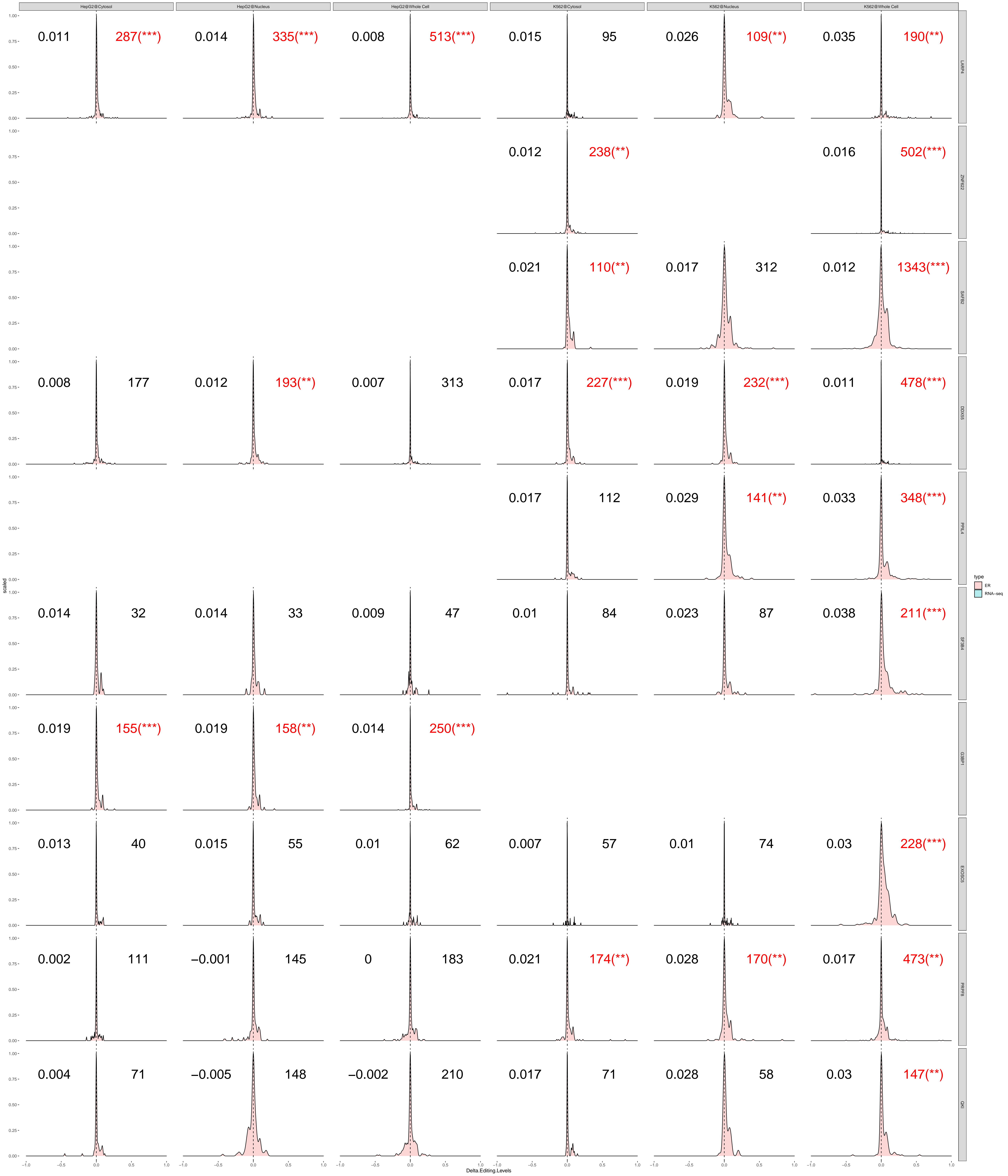

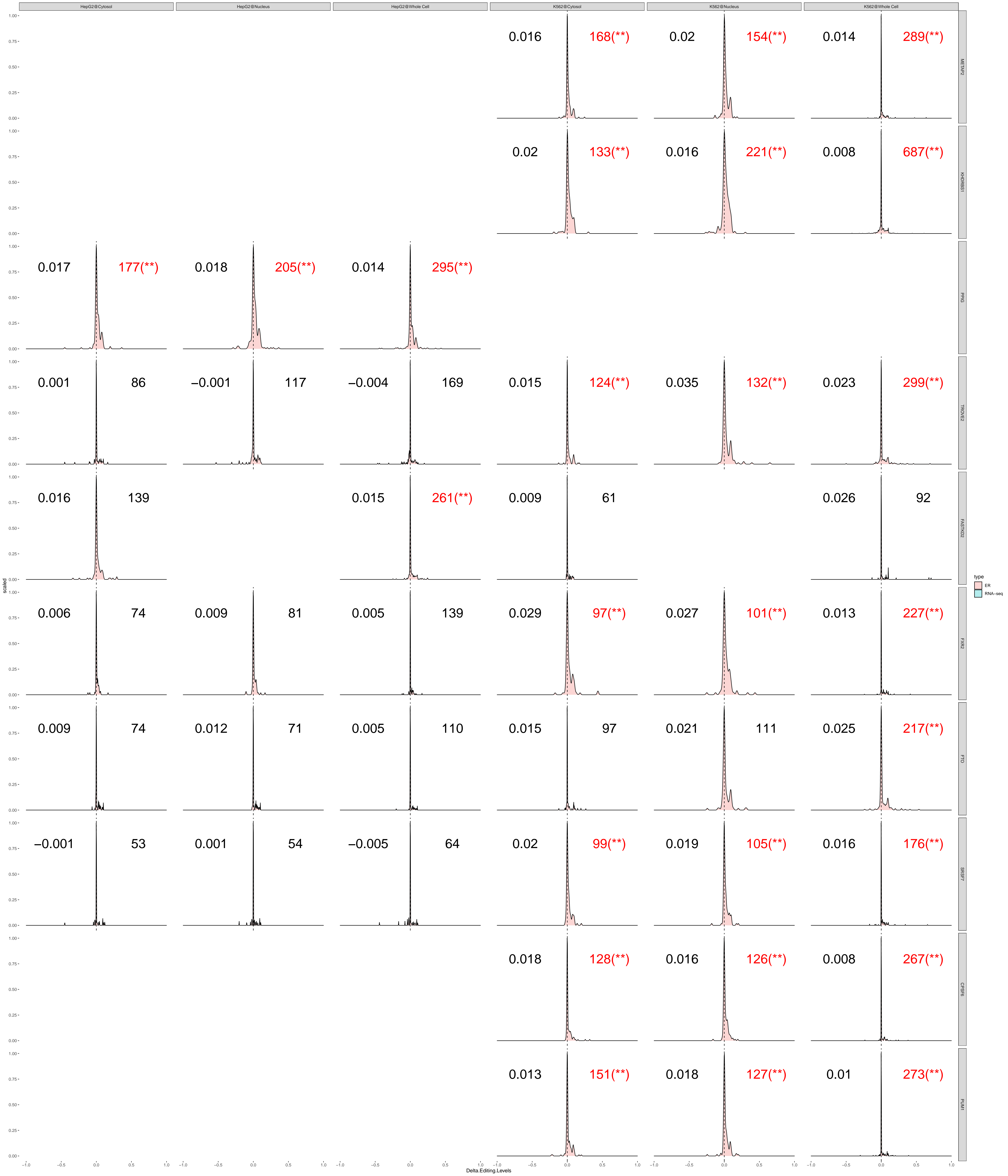

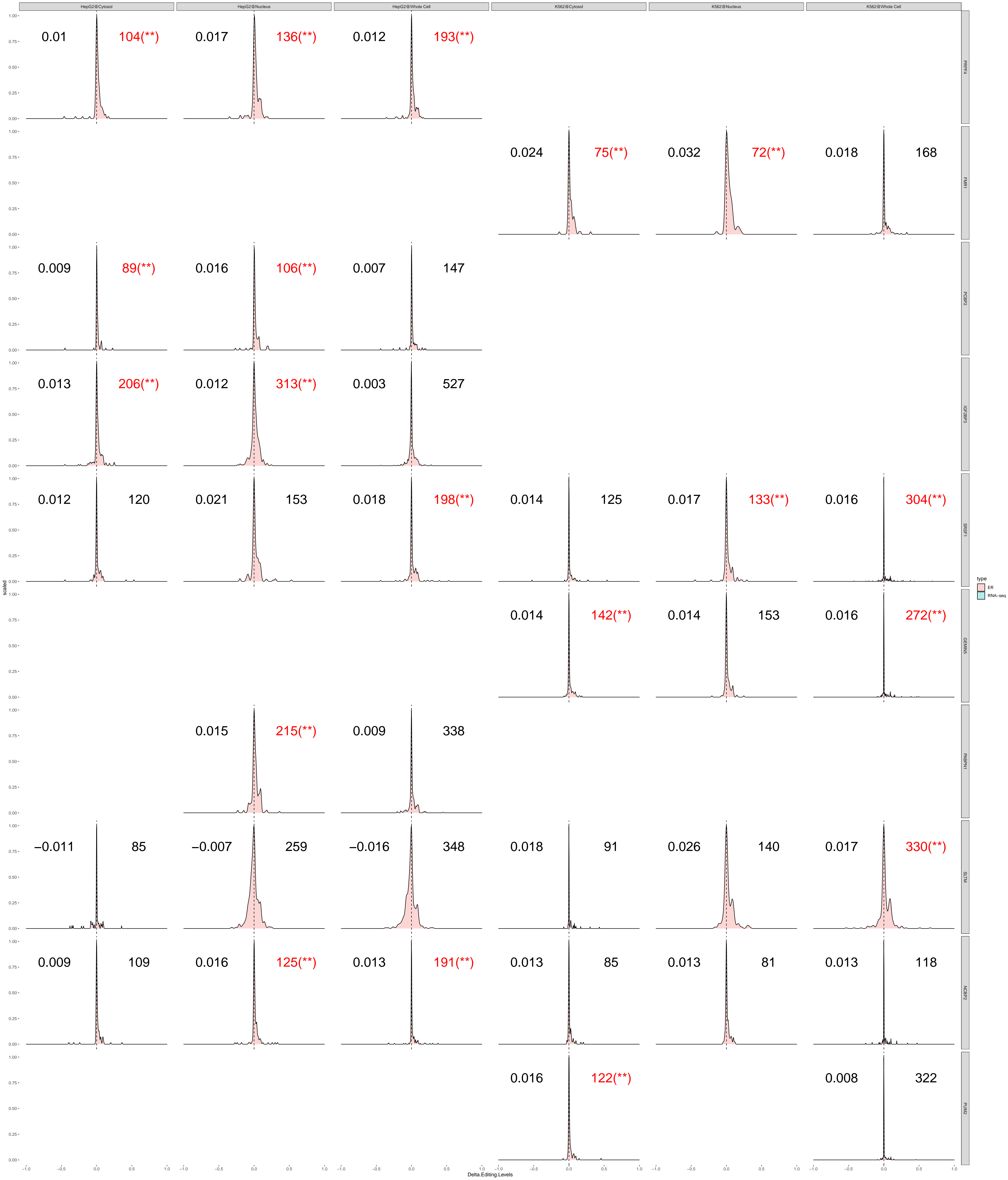

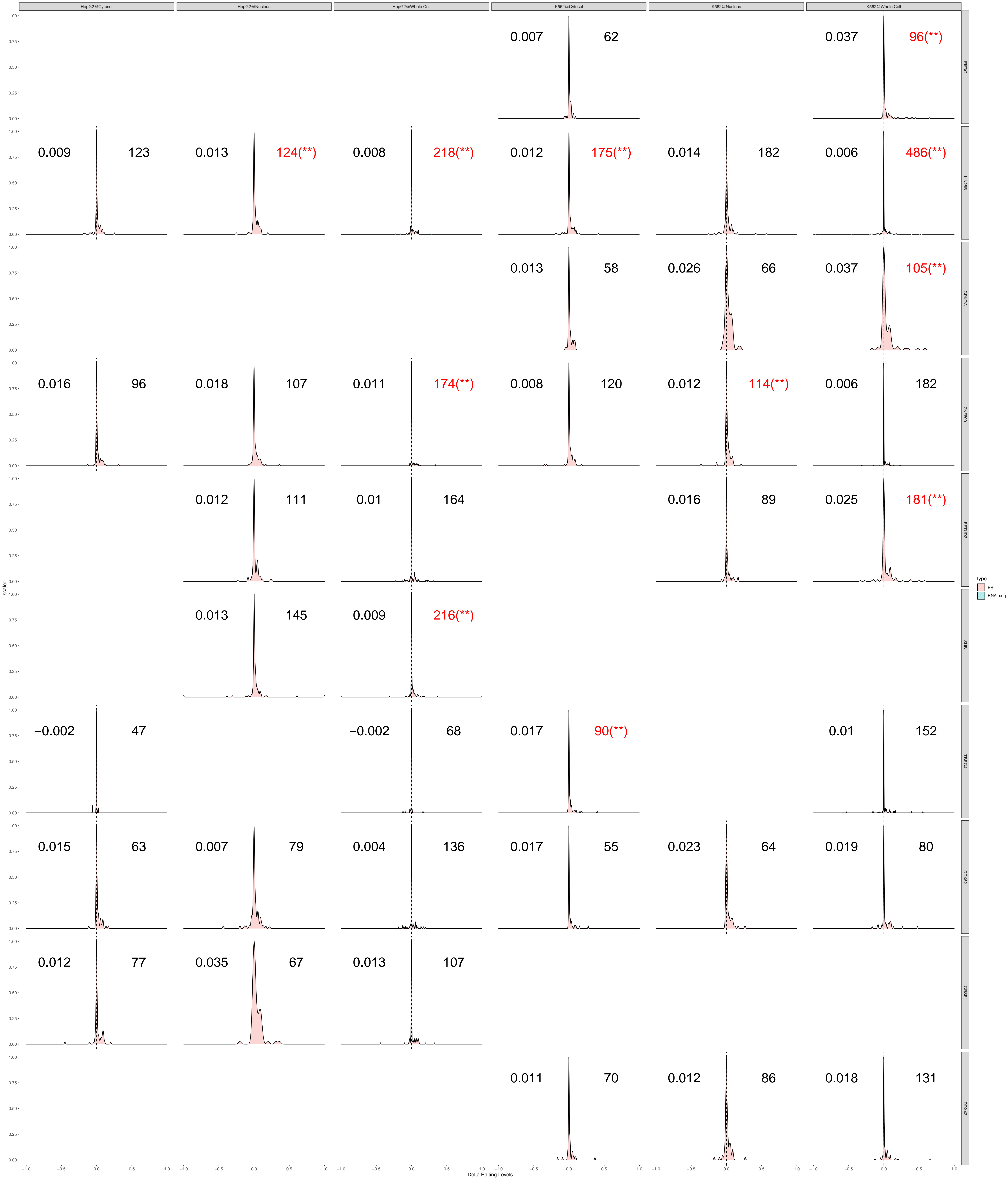

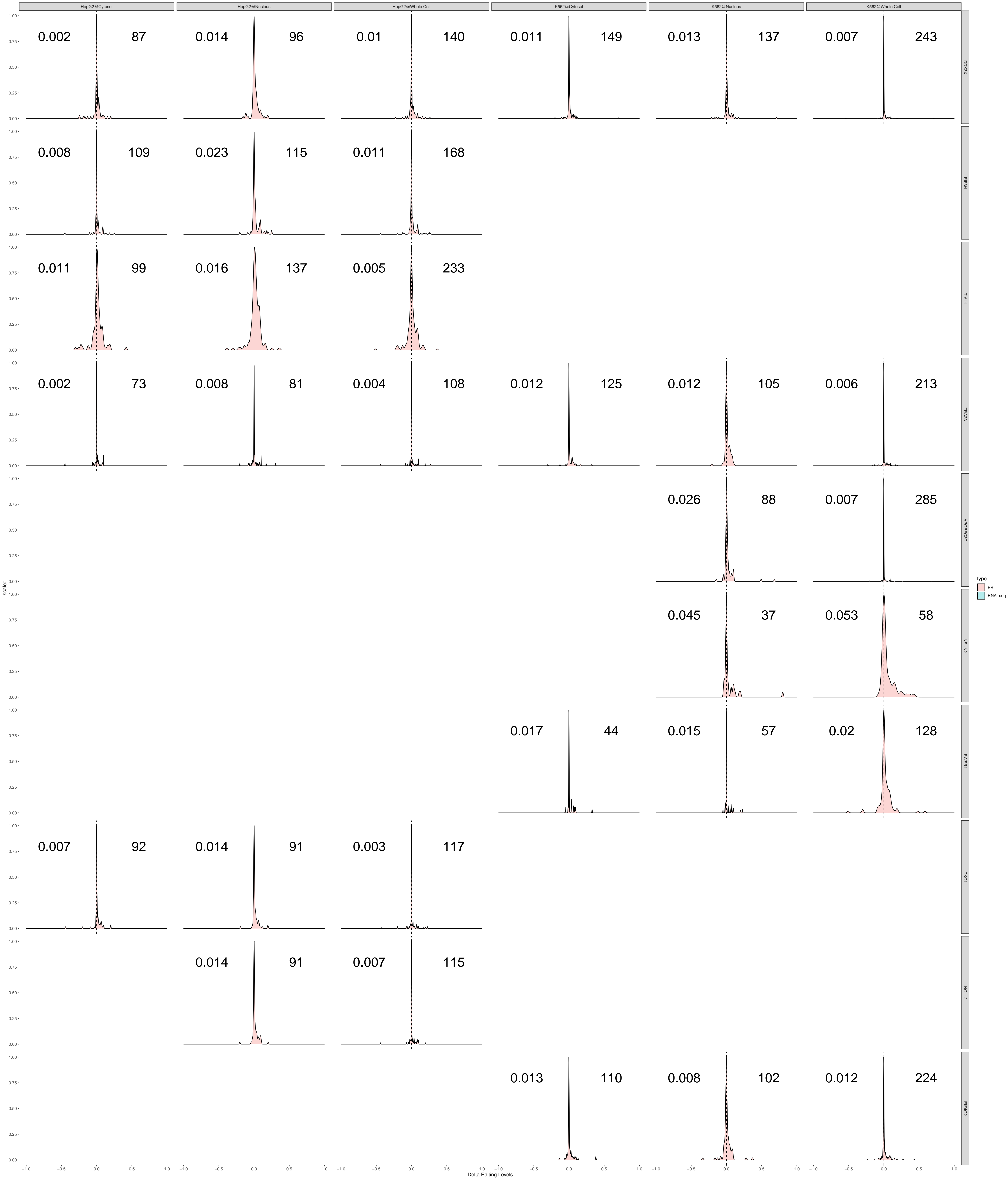

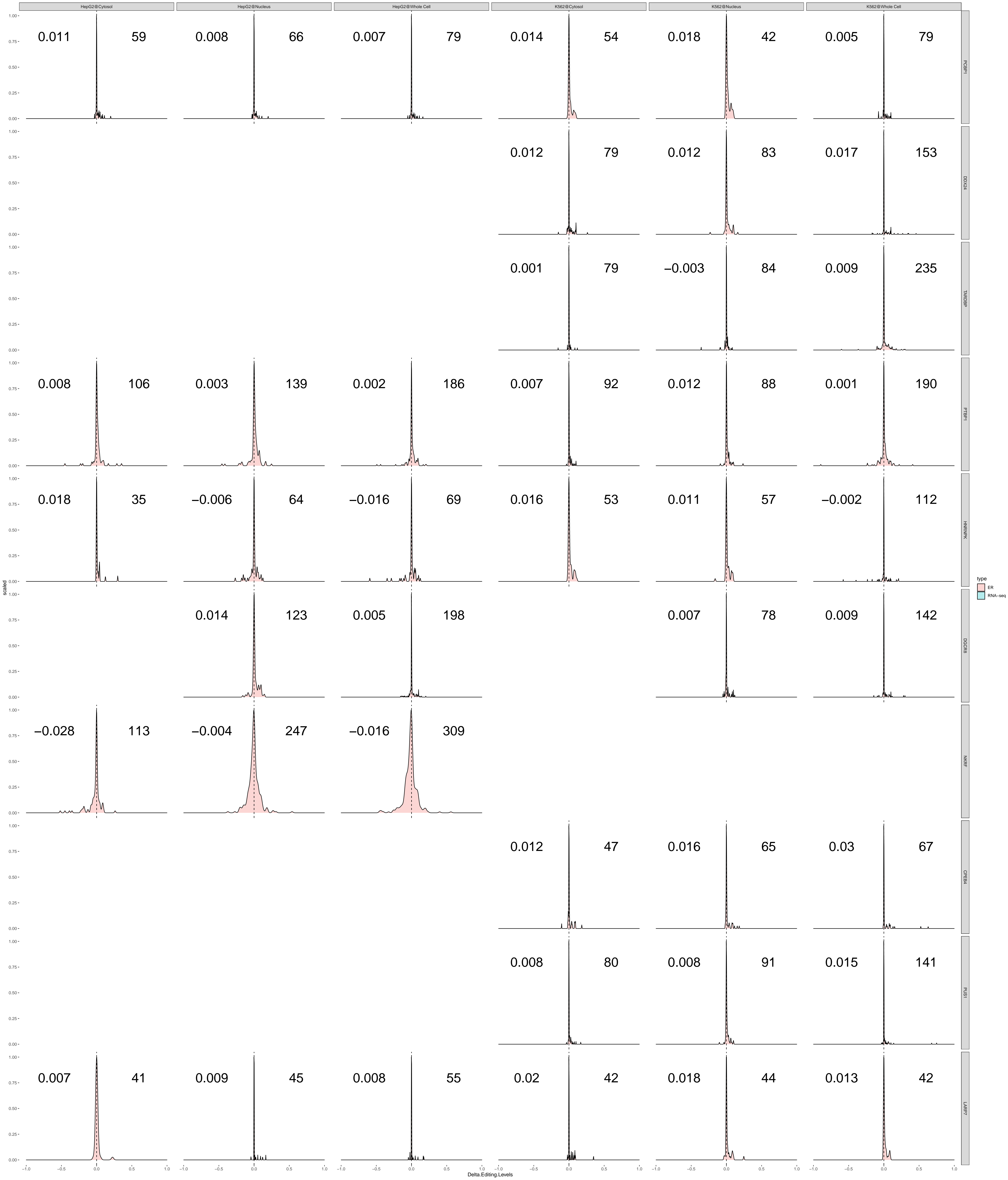

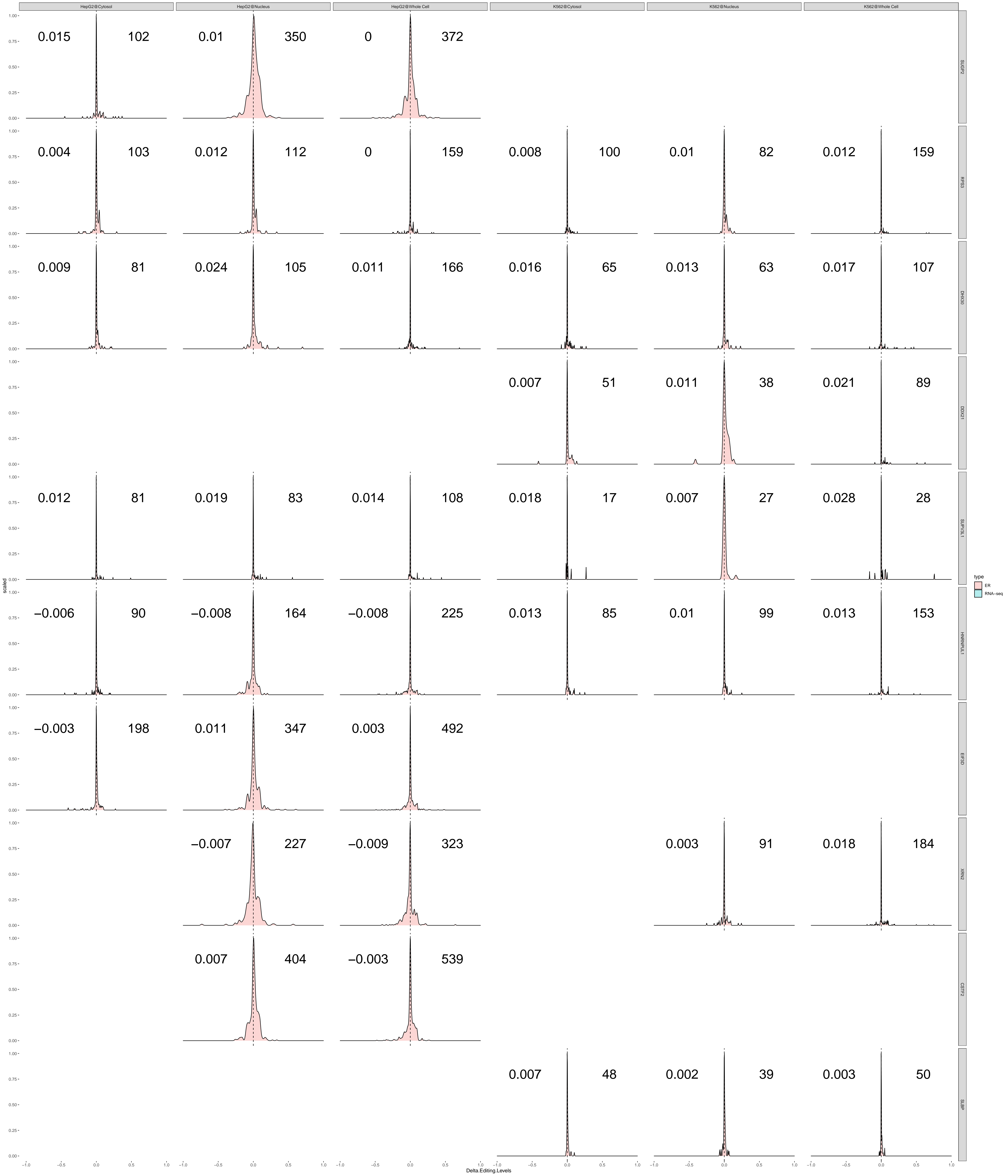

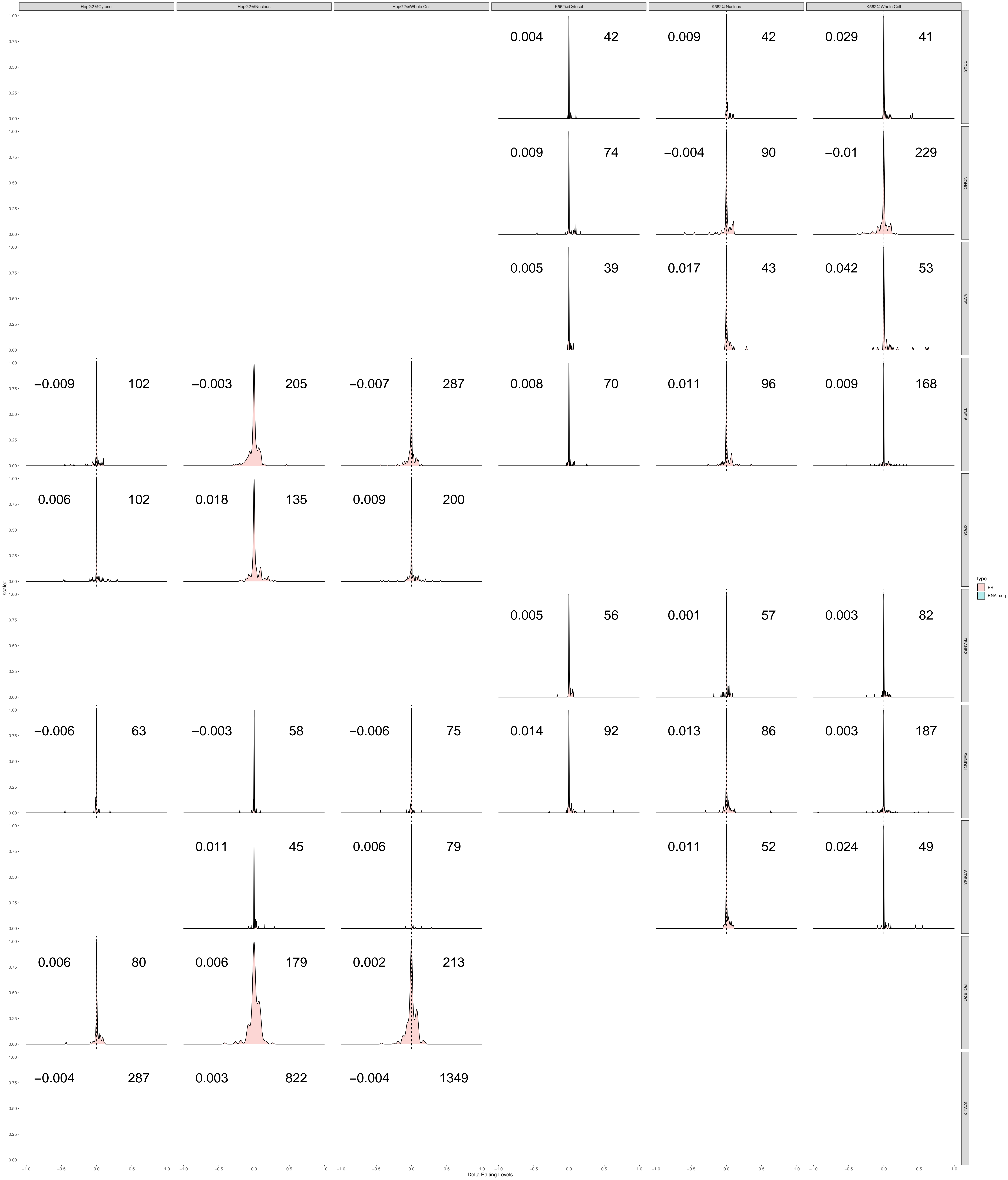

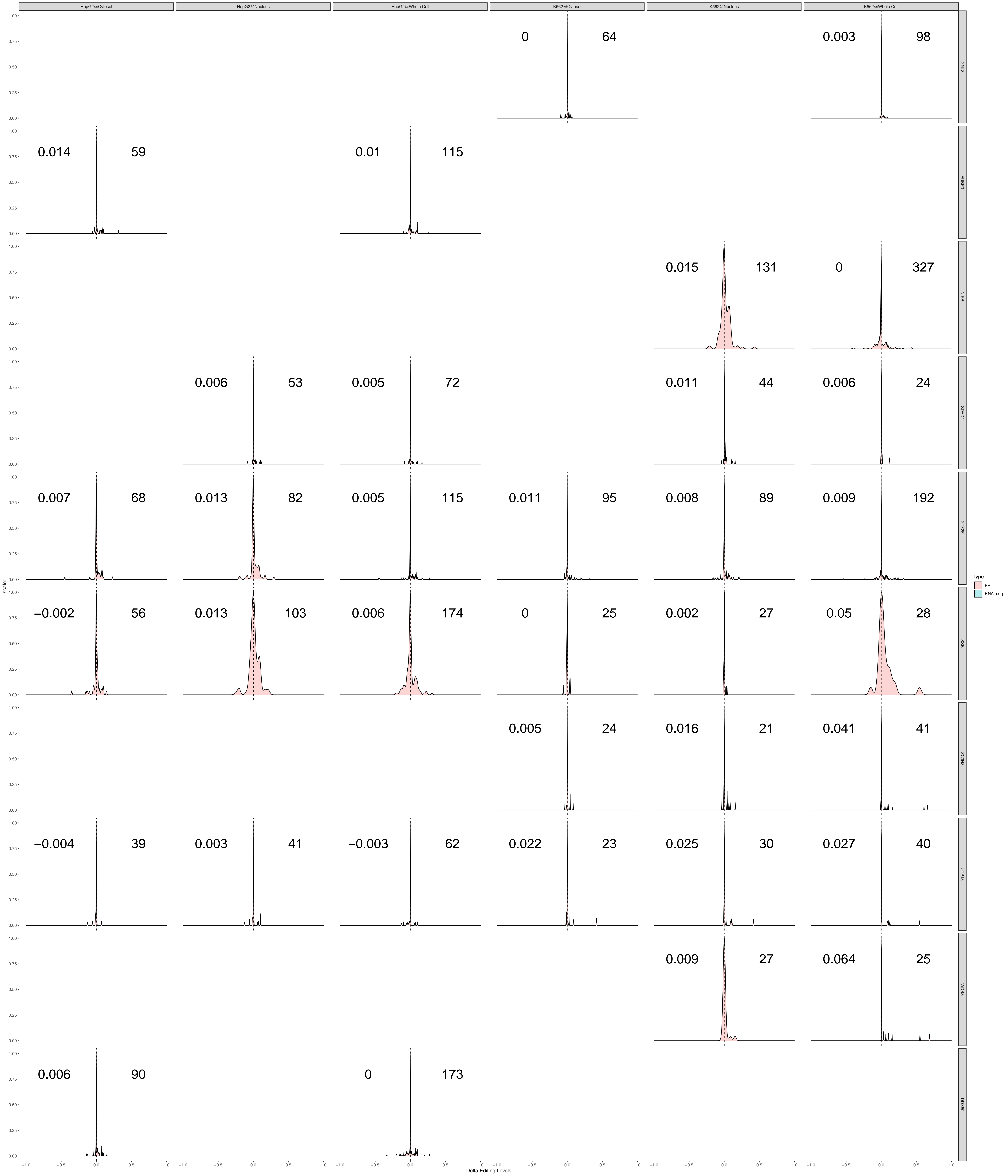

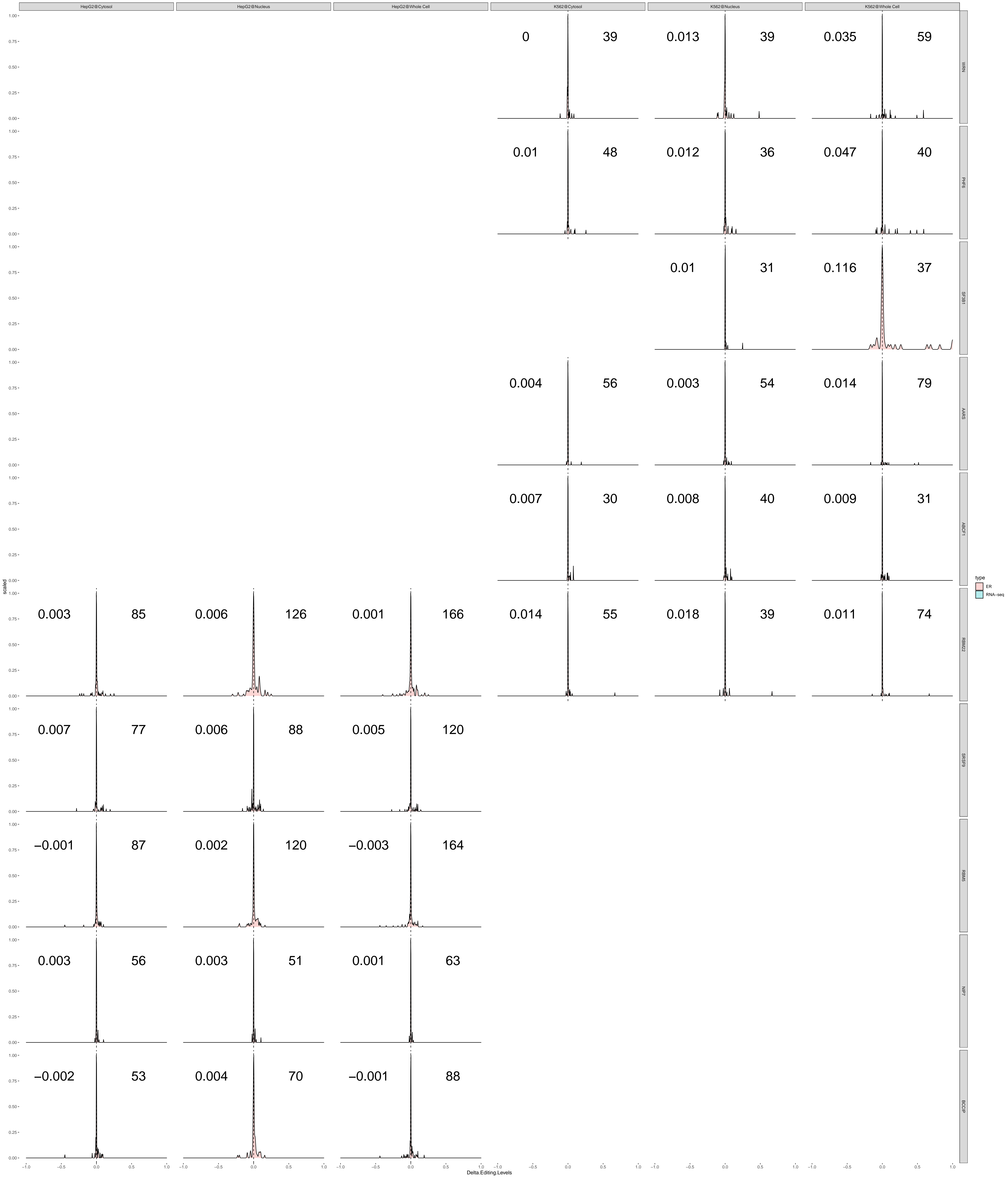

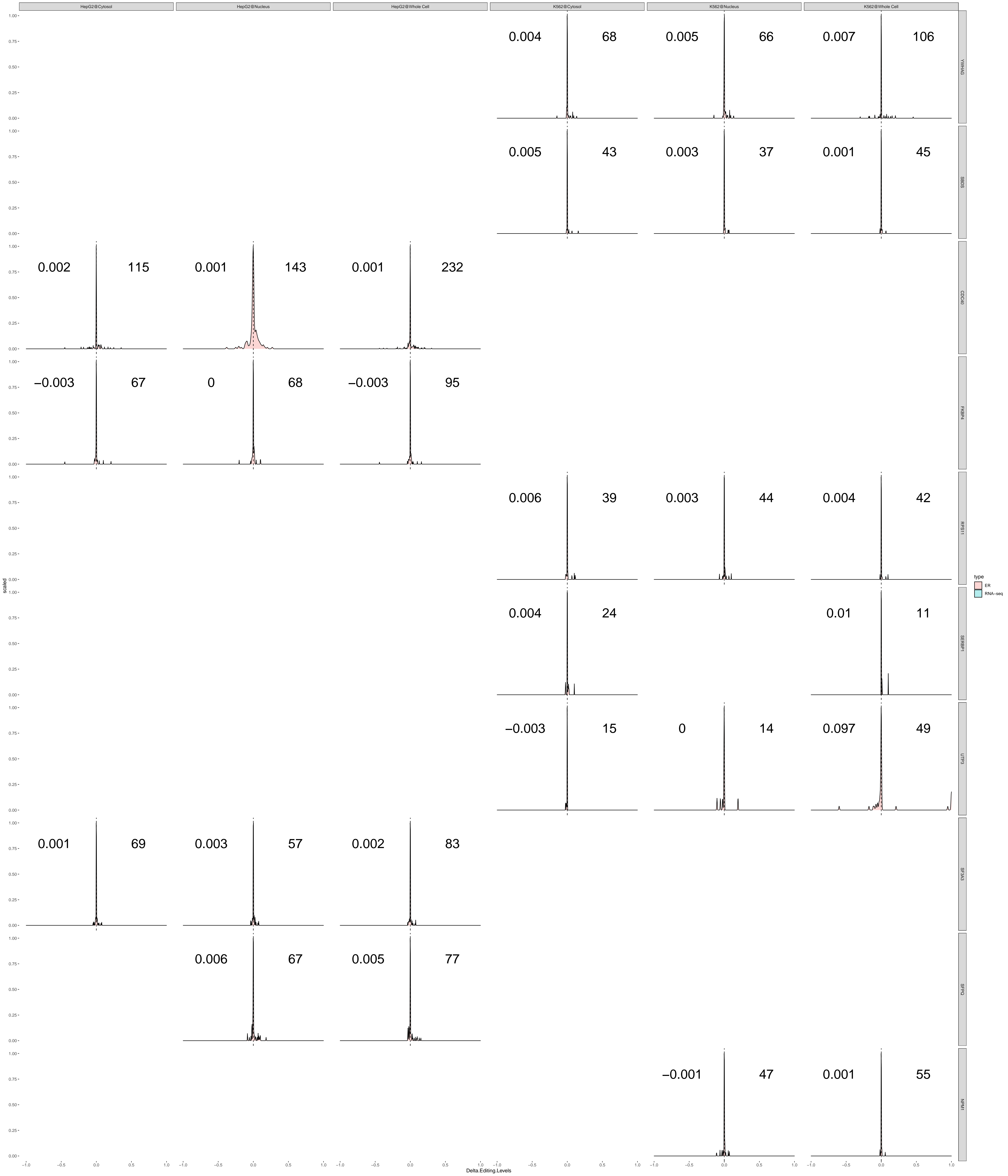

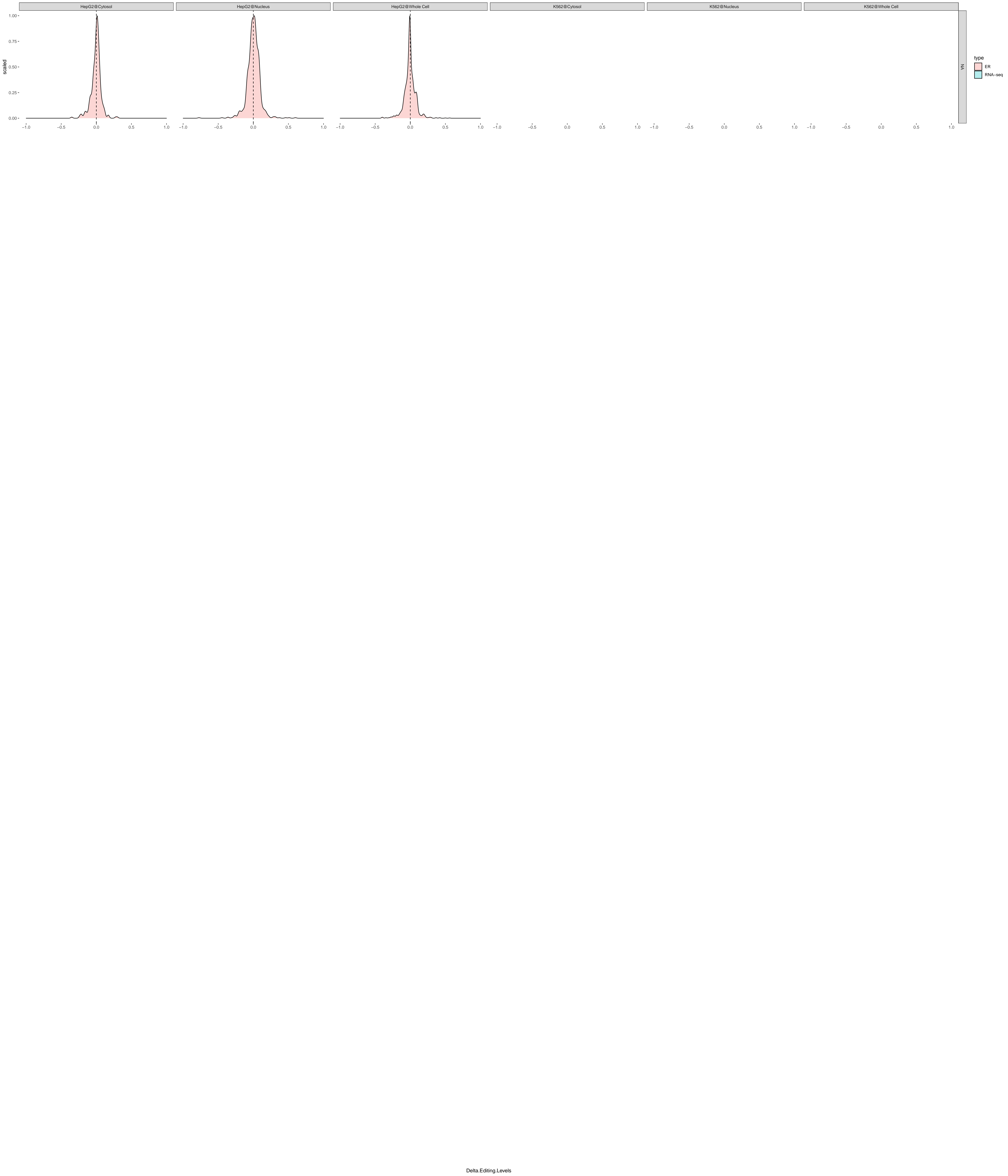

Supplement: Supplementary file 6 — Additional file 6: Distributions of RNA editing levels in RBP eCLIP and RNA-seq data. [file 13059_2022_2741_MOESM6_ESM.pdf]

Fig. 3

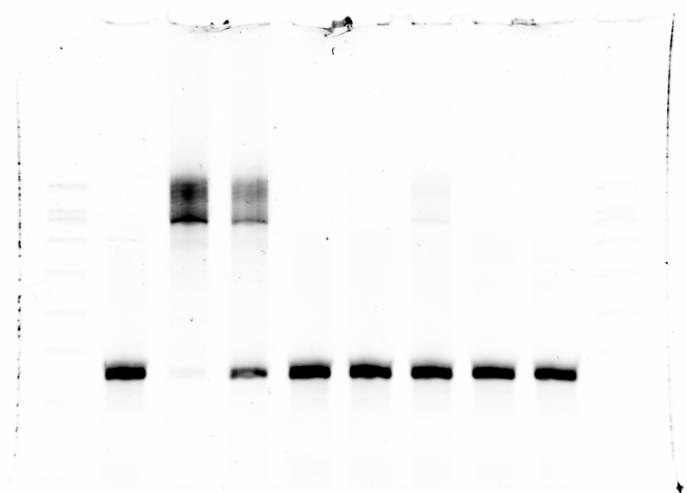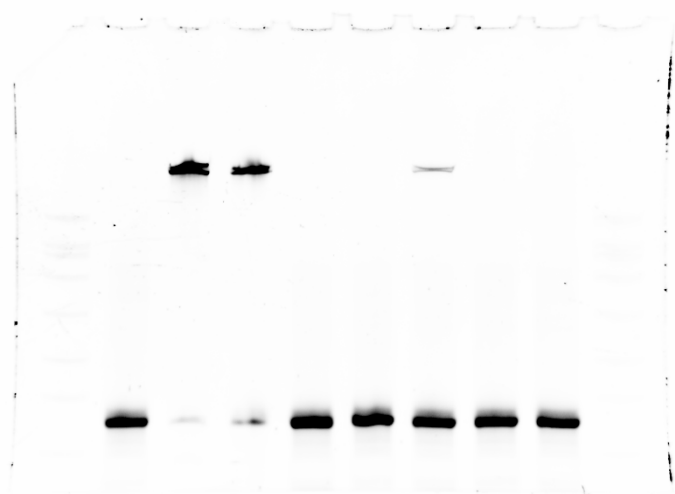

Fig. 4

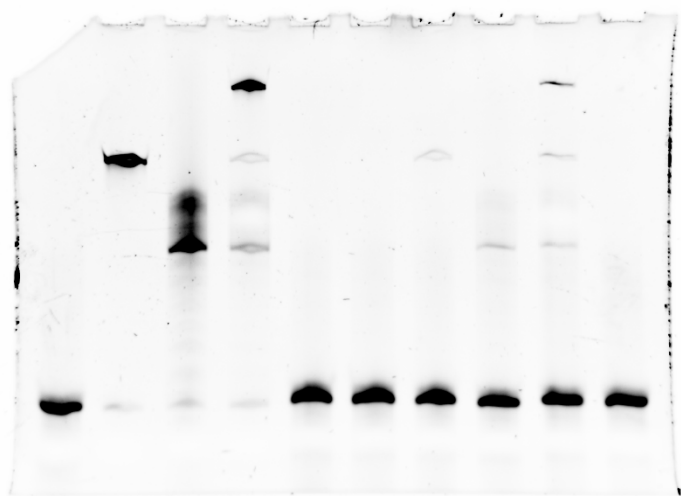

Fig. 6

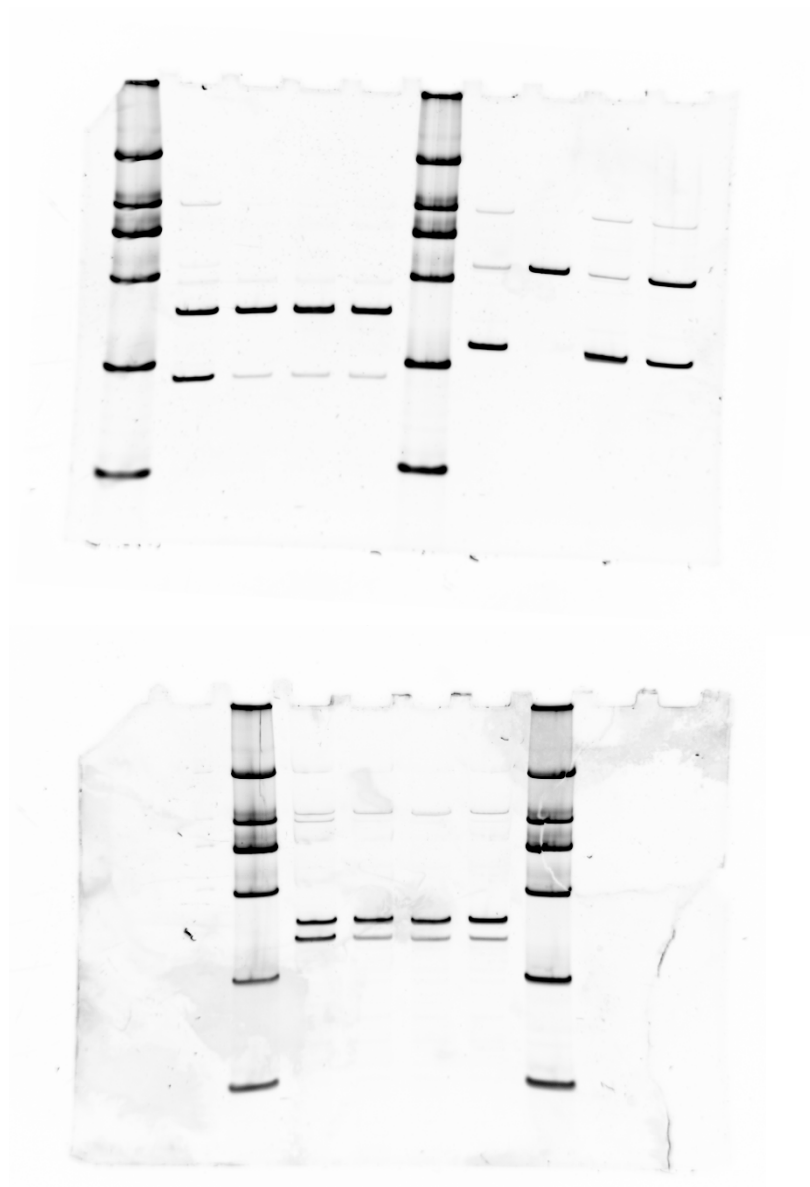

Supplement: Supplementary file 10 — Additional file 10. Uncropped images for the blots in figures 3, 4 and 6. [file 13059_2022_2741_MOESM10_ESM.pdf]
