# Supplementary material for: Survey of the binding preferences of RNA-binding proteins to RNA editing events
Source: Genome Biol. 2022 Aug 4;23:169. doi: 10.1186/s13059-022-02741-8 (PMC9351184; doi:10.1186/s13059-022-02741-8)
Supplement: Supplementary file 11 — Additional file 11. Review history. [file 13059_2022_2741_MOESM11_ESM.docx]

Review History

**First round of review**

**Reviewer 1**

**Were you able to assess all statistics in the manuscript, including the appropriateness of statistical tests used?**

Yes, and I have assessed the statistics in my report.

**Comments to author:**

In this manuscript, the authors cleverly reveal that some RBPs have significant binding preferences to specific RNA editing sites or edited sequences, based on RNA-seq and 150 RBP eCLIP-seq datasets in ENCODE. Further, they explore the relationship between RBP-related RNA editing events and RNA secondary structure or RNA splicing. These observations are complete and attractive. However, most of the conclusions are derived from bioinformatic analysis, and only partially are supported by experiments. For more accurate conclusions, complete quality control and optimized analysis for RNA editing events are necessary. Some inferences need to be supported by more data analysis or experimental results.

Major points:

1. Figure1 and S1: the A-to-I editing events were identified by RNA-seq datasets in K562 and HepG2 cells. But RNA editing sites and numbers of editing events varied widely between two cell lines, and among different cellular fractions. In addition to sequencing depth, other quality control results are essential: such as the proportion of A-to-I events and RNA editing motif.

2. Figure2 and 3: The binding preference of RBPs to RNA editing sites or edited sequences, was assessed by comparing the editing levels in the eCLIP-seq and RNA-seq. Here, the editing events were defined by both the RBP eCLIP and RNA-seq datasets. I have two concerns about this:

(1) Some editing events were supplemented by low coverage eCLIP-seq datasets, many of which may be false-positive editing events. Are eCLIP-seq data suitable for detecting RNA editing events?

(2) The eCLIP and RNA-seq datasets were generated from different laboratories and different sequencing platforms, which resulted in many editing events being detected only in the eCLIP datasets. The preference of RBP to specific RNA editing sites or edited sequences may be caused by the editing events detected only in eCLIP datasets.

3. Figure 3E: the authors speculated that strong binding preferences of the UPF1 or DROSHA to specific edited RNAs may be related to their function in controlling miRNA maturation, or RNA decay. Whether there is more bioinformatics analysis to support these inferences? For example, the expression changes of UPF1-associated edited RNA upon UPF1 knockdown.

4. Figure4: the strong interaction and multiple shared editing sites among UPF1, ILF3 and HNRNPC may be due to the abundance of RBP-associated editing events per RBP. It is suggested that the weight of the edges represents the degree of overlap between two RBPs' editing events, which is evaluated by "Jaccard Index", rather than the number of RBPs' shared editing events.

5. EMSA assays confirmed the preferable binding of both HNRNPC and ILF3 to the edited RNA. Dose the preferable binding affinities of these RBPs to edited RNA depend on the conserved sequence (such as HNRNPC motif) or RNA secondary structure?

Minor points:

1. The cell line names in Figures S1A and B seem to be reversed.

2. The cell line name is missed in Figure 3E results.

3. The resolution of the pictures in Figure S7b is too low to see the specific annotations.

4. Confused about the sum of RBP-associated RNA editing sites in Figure 6b. For example, there are 78 U2AF2-associated RNA editing sites in Figure 6B, but more than 100 sites are shown in Figure 3C.

5. The column name of column 19 seems to be "Total In RNA-seq", instead of "Total Alt In RNA-seq".

6. The definition of "RBP-associated editing sites" is unclear. In Figure 2, the "RBP-associated editing sites" represents the A-to-I RNA editing events detected in both the RBP eCLIP and RNA-seq data. However, in Figures 3 and S5, the "RBP-associated editing sites" represents the editing events favored or disfavored by RBPs.

**Authors Response**

**Point-by-point responses to the reviewers’ comments:**

Reviewer #1:

In this manuscript, the authors cleverly reveal that some RBPs have significant binding preferences to specific RNA editing sites or edited sequences, based on RNA-seq and 150 RBP eCLIP-seq datasets in ENCODE. Further, they explore the relationship between RBP-related RNA editing events and RNA secondary structure or RNA splicing. These observations are complete and attractive. However, most of the conclusions are derived from bioinformatic analysis, and only partially are supported by experiments. For more accurate conclusions, complete quality control and optimized analysis for RNA editing events are necessary. Some inferences need to be supported by more data analysis or experimental results.

*Response: We are delighted to see that the reviewer has found our work complete and attractive. We would like to thank the reviewer for the careful review and for providing very thoughtful comments and suggestions, which have greatly helped us further improve the manuscript.*

*This study is indeed mostly based on integrative analysis of public data, which were obtained from a large variety of experiments, including profiling of the RNA-binding target regions of 150 RBPs with eCLIP-seq, RNA-seq in the same two cells as background of the eCLIP-seq assays, profiling of RNA secondary structures with PARS-seq, profiling of alternative splicing upon knockdown of ADAR or other RBPs, etc. We then performed EMSA assays to validate the binding preferences of some RBPs to RNA editing events (Fig. 3D). In addition, we performed knock-down experiments to confirm some of the new insights from the data-mining results, i.e., the alternative RNA splicing patterns depend on UPF1-associated RNA editing events and the presence of the RBP UPF1 (Fig. 6D).*

*We have added more analyses and discussions to address the reviewer’s comments. Please refer to the following point-by-point responses for the details.*

Major points:

1. Figure1 and S1: the A-to-I editing events were identified by RNA-seq datasets in K562 and HepG2 cells. But RNA editing sites and numbers of editing events varied widely between two cell lines, and among different cellular fractions. In addition to sequencing depth, other quality control results are essential: such as the proportion of A-to-I events and RNA editing motif.

*Response: We are sorry for not describing the process for identification of the editing events more clearly. Only the A-to-I editing events were considered, and they were identified from RNA-seq and eCLIP-seq datasets, by using REDItools [1] based on the RNA editing database REDIportal [2]. Established by the GTEx project, REDIportal is an archive of the previously annotated RNA-editing events in 9642 human RNA-seq samples from 549 individuals [3]. Next, the dbSNP [4] database was used to further remove the apparent RNA editing events that are potentially due to SNPs. Thus, all the RNA-editing events from either the RNA-seq or the eCLIP-seq data in this study are previously known and already annotated as RNA-editing events. We believe that this whole process above has largely eliminated the potential false discoveries of RNA editing. The Methods section has been updated to better describe the process above (page 24-25).*

*As the reviewer has noted, the RNA-editing events vary widely between the two cell lines, which is well in line with the previously reported strong cell type specificity of RNA editing [5-8]. The variations between different cell fractions are also consistent with previous understandings about the subcellular preferences of the different RNA editing events. First, the intracellular locations of the RNA molecules are highly unbalanced between the nucleus and the cytosol. 90% of the nucleotides are spliced out as introns, and less than 10% of the nucleotides kept in the mature RNA [9]. It is estimated that only about 30% of the RNA transcripts are processed and exported to the cytoplasm [10]. Second, the RNA-editing events carried out by ADAR P110 in the nucleus are mostly in the introns, which are spliced out and kept in the nucleus. On the other hand, the RNA-editing events carried out by ADAR P150 in the cytosol should be mostly kept in the cytosol and therefore undetectable in the nucleus [11]. Finally, it is worth noting that for the RNA editing sites detected in multiple different cellular fractions (nucleus, cytoplasm, and whole cell), the editing levels of the majority are mostly consistent across different fractions (Fig. 1B). Very few RNA editing sites have significantly different editing levels across different fractions (Fig. 1B), and these sites have been removed for further analyses.*

*The majority of the RNA-editing events identified in our study are located within the Alu regions (Fig. S1A), which is consistent with previous studies of RNA editing [12, 13]. The RNA editing events are known to have a weak motif of U/A/C[A]S (‘S’ for strong G) [14-16]. As shown in the following figure (Fig. S1B), such a motif is also observed in our results, which is highly consistent with previous reports [14-16]. These QC results have been added in Fig. S1A, B, and introduced on page 4-5 in the revised manuscript.*

2. Figure2 and 3: The binding preference of RBPs to RNA editing sites or edited sequences, was assessed by comparing the editing levels in the eCLIP-seq and RNA-seq. Here, the editing events were defined by both the RBP eCLIP and RNA-seq datasets. I have two concerns about this:

(1) Some editing events were supplemented by low coverage eCLIP-seq datasets, many of which may be false-positive editing events. Are eCLIP-seq data suitable for detecting RNA editing events?

*Response: We thank the reviewer for raising this question. As discussed in our response to the first comment, all the editing events were identified from RNA-seq or eCLIP-seq data by using the previously annotated RNA editing sites in REDIportal [2] as a reference. The dbSNP database [4] was then used to filter out the RNA editing events that are potentially due to SNPs. Therefore, as discussed above, we only used the previously annotated RNA editing events rather than identifying novel RNA editing events from eCLIP-seq data.*

*In addition, only the RNA editing sites with more than 10 reads were used for the down-stream analyses. Importantly, the reads were counted after removing the PCR duplicates. This is a common practice in previous studies of RNA editing [17-21]. Therefore, although eCLIP-seq data may not be suitable for detecting new editing events, we believe that it can be used to quantify the editing levels for the annotated RNA editing sites with reasonable read densities.*

(2) The eCLIP and RNA-seq datasets were generated from different laboratories and different sequencing platforms, which resulted in many editing events being detected only in the eCLIP datasets. The preference of RBP to specific RNA editing sites or edited sequences may be caused by the editing events detected only in eCLIP datasets.

*Response: As discussed above, the eCLIP-seq or RNA-seq data was not used to detect new editing events per se. They were essentially used to estimate the editing levels of previously annotated RNA editing sites in regular RNA samples or in RBP-associated RNA samples. The eCLIP and RNA-seq datasets were indeed generated with different library preparation protocols. In fact, even if they were generated from the same lab, these two types of data would still be different in many ways. However, our analysis calculates the editing levels, i.e., percentage of the reads with A-to-I conversion in the total reads, and evaluates the statistical significance of the editing level differences by comparing the RNA-seq and eCLIP-seq data. Therefore, we believe that as long as the RNA-seq or eCLIP-seq protocols do not have strong bias for the edited or unedited RNA, the editing levels estimated from these two types of data should be comparable. In fact, to address the reviewer’s question, as Fig. 3E shows, the majority of the RBP-associated RNA editing sites have non-zero editing levels in both RNA-seq and eCLIP-seq data. Therefore, for most cases of the RBP-favoring RNA editing events, these editing events were in fact detected in both types of data, not only in the eCLIP-seq data.*

*Specifically, to estimate the editing levels, we first filtered out the editing sites with fewer than 10 reads in either the RNA-seq or eCLIP-seq data, after removing the PCR duplicates. Previous studies have also used similar criteria to identify the events of differential RNA editing by comparing different RNA-seq datasets [17-21]. Next, to compare the editing levels between different datasets (i.e., eCLIP-seq vs. RNA-seq), we used the Fisher’s exact test [22], which takes into account the read densities to evaluate the statistical significance of the editing level difference. In other words, for the sites with relatively low read coverages, much larger differences of the editing levels would be needed to be deemed as statistically significant. For example, for a site with 10 RNA-seq reads of A and zero read of I (editing ratio of 0/10), the eCLIP-seq data would need to show at least 4/10 (4 out of 10 reads), or 5/11, etc. to be called as significant in our results. We believe that from the statistical and biological points of view, such differences are large enough to be biologically relevant.*

3. Figure 3E: the authors speculated that strong binding preferences of the UPF1 or DROSHA to specific edited RNAs may be related to their function in controlling miRNA maturation, or RNA decay. Whether there is more bioinformatics analysis to support these inferences? For example, the expression changes of UPF1-associated edited RNA upon UPF1 knockdown.

*Response: We appreciate the reviewer’s question. As discussed in the manuscript on page 10, the association between the ADAR-mediated A-to-I RNA editing and the UPF1-mediated RNA degradation has been well established before [23]. This previous study showed that the A-to-I editing could serve as an RNA surveillance machinery, which is aided by RNA degradation executed by UPF1. Here, our analysis further showed strong preferences of UPF1 to the edited RNA. Considering the previous reports and our observations here, we think it is a reasonable speculation that the editing level of the RNA sequence could be at play in UPF1-mediated RNA degradation. The text of the manuscript has been modified to better convey the message here (page 10).*

*As discussed on page 10, DROSHA has been shown to promote RNA editing, potentially via direct interaction with ADAR1 [6]. On the other hand, ADAR1 enhances miRNA maturation [24]. Our analysis has revealed a strong preference of DROSHA to RNA editing events, but we did not find a clear and simple connection between the DROSHA-mediated miRNA maturation and the DROSHA-associated RNA editing events. We thank the reviewer for raising this question, which is certainly worth further in-depth investigation in the future.*

4. Figure4: the strong interaction and multiple shared editing sites among UPF1, ILF3 and HNRNPC may be due to the abundance of RBP-associated editing events per RBP. It is suggested that the weight of the edges represents the degree of overlap between two RBPs' editing events, which is evaluated by "Jaccard Index", rather than the number of RBPs' shared editing events.

*Response: We totally agree with the reviewer that the Jaccard Index, which takes into account the sizes of the two sets, is more commonly used for evaluation of the overlap. However, here we used the numbers of shared editing sites between two RBPs simply to query whether the RBPs are potentially associated by preferably binding to the same set of RNA editing sites. We think that the absolute numbers reflect the potential functional associations better.*

*Hypothetically, two RBPs could have a small Jaccard Index due to large numbers of RNA editing events associated to each of them. However, if they do share a relatively large number of the editing events, we think that it is reasonable to grant a high priority for the potential functional association between these two RBPs. On the other hand, two RBPs sharing a small set of RNA editing events could still have a large Jaccard Index, simply due to the small scales of their associations to the RNA editing events. We think that these cases should be de-prioritized. In other words, we believe it is the absolute numbers of the shared editing events that matter in establishing the potential functional associations between RBPs. Therefore, we prefer to stick to the numbers of the shared editing events as weights of the RBP associations. A sentence has been added in the revised manuscript to explain for this choice (page 12).*

5. EMSA assays confirmed the preferable binding of both HNRNPC and ILF3 to the edited RNA. Dose the preferable binding affinities of these RBPs to edited RNA depend on the conserved sequence (such as HNRNPC motif) or RNA secondary structure?

*Response: We appreciate the reviewer’s interesting question, which indeed worth discussion in the manuscript. The preferable binding of RBPs to the edited or unedited RNA could depend on either the RNA sequence or the RNA secondary structure, varying for different RBPs. RNA editing leads to changes of the RNA sequence itself, and it is also well acknowledged that such changes of the RNA sequence usually result in shifts of the RNA secondary structures as well [25-28]. Therefore, it is quite likely that some RBPs favorably binds to the edited or unedited RNA due to the RBP-binding sequence motifs, while some other RBPs recognize the RNA secondary structure formed by the edited or unedited RNA.*

*ILF3 is a well-appreciated dsRNA-binding protein [29]. Interestingly, it has been further shown that ILF3, like ADAR2, binds to certain dsRNA structures with specific sequence features [30]. In other words, both the dsRNA structure and the underlying sequence together determine the binding of ILF3 to the edited RNA. For HNRNPC, like the reviewer has mentioned, its RNA binding depends on HNRNPC recognition motifs such as uridine (U)-rich sequences rather than specific secondary structures [31].*

*In summary, the EMSA assays can not reveal whether the RBP’s preferences to the RNA editing events depend on the RNA sequence motifs or the secondary structures. It is usually hard to precisely distinguish the influence of RNA sequence context and RNA secondary structure to an event of RBP binding. However, as discussed above, according to the extensive studies before, we highly suspect that the binding preference of ILF3 to the RNA editing sites depends on both the sequence and the structure, whereas the preference of HNRNPC depends more on the RNA sequence itself. The discussion above has been added in the revised manuscript (page 20-21).*

Minor points:

1. The cell line names in Figures S1A and B seem to be reversed.

*Response: We are sorry the mistake, and we thank the reviewer for pointing it out. The cell line names have been corrected.*

2. The cell line name is missed in Figure 3E results.

*Response: We thank the reviewer for the reminder. We have added the cell line name in Fig. 3E.*

3. The resolution of the pictures in Figure S7b is too low to see the specific annotations.

*Response: We have replaced Fig. S7B with a new image with higher resolution.*

4. Confused about the sum of RBP-associated RNA editing sites in Figure 6b. For example, there are 78 U2AF2-associated RNA editing sites in Figure 6B, but more than 100 sites are shown in Figure 3C.

*Response: We are sorry for the confusion due to misleading terms. Fig. 6B was designated to discuss about the potential associations between the RBP-associated RNA editing events and alternative splicing. Therefore, only the RNA editing sites falling inside gene bodies were considered. Some RNA editing sites were in the regions annotated as intergenic, thereby not being included in Fig. 6B. However, Fig. 3C showed all the RNA editing sites. We now have made clear this extra layer of filter for Fig. 6B in the main text, figure legend, and in the figure itself (page 17, 19 20).*

5. The column name of column 19 seems to be "Total In RNA-seq", instead of "Total Alt In RNA-seq".

*Response: We thank the reviewer for pointing this out. The column name has been corrected in all the supplementary tables.*

6. The definition of "RBP-associated editing sites" is unclear. In Figure 2, the "RBP-associated editing sites" represents the A-to-I RNA editing events detected in both the RBP eCLIP and RNA-seq data. However, in Figures 3 and S5, the "RBP-associated editing sites" represents the editing events favored or disfavored by RBPs.

*Response: We are sorry for the misleading terms. Indeed, the term of “RBP-associated editing sites” represents the editing events favored or disfavored by RBPs. The misleading terms of “RBP-associated editing sites” used for the discussion of Fig. 2 have been changed to “RBP-bound editing sites” (page 7).*

*References*

*1. Picardi, E. and Pesole, G., REDItools: high-throughput RNA editing detection made easy. Bioinformatics, 2013. 29(14): p. 1813-1814.*

*2. Picardi, E., D'Erchia, A.M., Lo Giudice, C., and Pesole, G., REDIportal: a comprehensive database of A-to-I RNA editing events in humans. Nucleic acids research, 2017. 45(D1): p. D750-D757.*

*3. Consortium, G., The Genotype-Tissue Expression (GTEx) pilot analysis: multitissue gene regulation in humans. Science, 2015. 348(6235): p. 648-660.*

*4. Sherry, S.T., Ward, M.-H., Kholodov, M., Baker, J., Phan, L., Smigielski, E.M., and Sirotkin, K., dbSNP: the NCBI database of genetic variation. Nucleic acids research, 2001. 29(1): p. 308-311.*

*5. Tan, M.H., Li, Q., Shanmugam, R., Piskol, R., Kohler, J.N., Young, A.N., Liu, K.I., Zhang, R., Ramaswami, G., and Ariyoshi, K., Dynamic landscape and regulation of RNA editing in mammals. Nature, 2017. 550(7675): p. 249-254.*

*6. Quinones-Valdez, G., Tran, S.S., Jun, H.I., Bahn, J.H., Yang, E.W., Zhan, L., Brummer, A., Wei, X., Van Nostrand, E.L., Pratt, G.A., Yeo, G.W., Graveley, B.R., and Xiao, X., Regulation of RNA editing by RNA-binding proteins in human cells. Commun Biol, 2019. 2: p. 19.*

*7. Han, L., Diao, L., Yu, S., Xu, X., Li, J., Zhang, R., Yang, Y., Werner, H.M.J., Eterovic, A.K., and Yuan, Y., The Genomic Landscape and Clinical Relevance of A-to-I RNA Editing in Human Cancers. Cancer Cell, 2015. 28(4): p. 515-528.*

*8. Picardi, E., Horner, D.S., and Pesole, G., Single-cell transcriptomics reveals specific RNA editing signatures in the human brain. Rna, 2017. 23(6): p. 860-865.*

*9. Liu, H., Luo, M., and Wen, J., mRNA stability in the nucleus. Journal of Zhejiang University-science B, 2014. 15(5): p. 444-454.*

*10. Jackson, D.A., Pombo, A., and Iborra, F.J., The balance sheet for transcription: an analysis of nuclear RNA metabolism in mammalian cells. The FASEB Journal, 2000. 14(2): p. 242-254.*

*11. Galipon, J., Ishii, R., Suzuki, Y., Tomita, M., and Ui-Tei, K., Differential binding of three major human ADAR isoforms to coding and long non-coding transcripts. Genes, 2017. 8(2): p. 68.*

*12. Quinones-Valdez, G., Tran, S.S., Jun, H.-I., Bahn, J.H., Yang, E.-W., Zhan, L., Brümmer, A., Wei, X., Van Nostrand, E.L., and Pratt, G.A., Regulation of RNA editing by RNA-binding proteins in human cells. Communications biology, 2019. 2(1): p. 1-14.*

*13. Schaffer, A.A. and Levanon, E.Y., ALU A-to-I RNA Editing: Millions of sites and many open questions. RNA Editing, 2021: p. 149-162.*

*14. Sun, T., Yu, Y., Wu, X., Acevedo, A., Luo, J.-D., Wang, J., Schneider, W.M., Hurwitz, B., Rosenberg, B.R., and Chung, H., Decoupling expression and editing preferences of ADAR1 p150 and p110 isoforms. Proceedings of the National Academy of Sciences, 2021. 118(12).*

*15. Bahn, J.H., Ahn, J., Lin, X., Zhang, Q., Lee, J.-H., Civelek, M., and Xiao, X., Genomic analysis of ADAR1 binding and its involvement in multiple RNA processing pathways. Nature communications, 2015. 6(1): p. 1-13.*

*16. Ishiguro, S., Galipon, J., Ishii, R., Suzuki, Y., Kondo, S., Okada-Hatakeyama, M., Tomita, M., and Ui-Tei, K., Base-pairing probability in the microRNA stem region affects the binding and editing specificity of human A-to-I editing enzymes ADAR1-p110 and ADAR2. RNA biology, 2018. 15(7): p. 976-989.*

*17. Van Nostrand, E.L., Freese, P., Pratt, G.A., Wang, X., Wei, X., Xiao, R., Blue, S.M., Chen, J.-Y., Cody, N.A., and Dominguez, D., A large-scale binding and functional map of human RNA-binding proteins. Nature, 2020. 583(7818): p. 711-719.*

*18. Bakhtiarizadeh, M.R., Salehi, A., and Rivera, R.M., Genome-wide identification and analysis of A-to-I RNA editing events in bovine by transcriptome sequencing. PLOS ONE, 2018. 13(2): p. e0193316.*

*19. Lo Giudice, C., Silvestris, D.A., Roth, S.H., Eisenberg, E., Pesole, G., Gallo, A., and Picardi, E., Quantifying RNA Editing in Deep Transcriptome Datasets. Frontiers in Genetics, 2020. 11(194).*

*20. Lo Giudice, C., Tangaro, M.A., Pesole, G., and Picardi, E., Investigating RNA editing in deep transcriptome datasets with REDItools and REDIportal. Nature Protocols, 2020. 15(3): p. 1098-1131.*

*21. Zhang, Y., Zhang, L., Yue, J., Wei, X., Wang, L., Liu, X., Gao, H., Hou, X., Zhao, F., Yan, H., and Wang, L., Genome-wide identification of RNA editing in seven porcine tissues by matched DNA and RNA high-throughput sequencing. Journal of Animal Science and Biotechnology, 2019. 10(1): p. 24.*

*22. Fisher, R.A., Statistical methods for research workers. Statistical methods for research workers., 1934(5th Ed).*

*23. Agranat, L., Raitskin, O., Sperling, J., and Sperling, R., The editing enzyme ADAR1 and the mRNA surveillance protein hUpf1 interact in the cell nucleus. Proceedings of the National Academy of Sciences of the United States of America, 2008. 105(13): p. 5028-5033.*

*24. Bahn, J.H., Ahn, J., Lin, X., Zhang, Q., Lee, J., Civelek, M., and Xiao, X., Genomic analysis of ADAR1 binding and its involvement in multiple RNA processing pathways. Nature Communications, 2015. 6(1): p. 6355-6355.*

*25. Hundley, H.A. and Bass, B.L., ADAR editing in double-stranded UTRs and other noncoding RNA sequences. Trends Biochem Sci, 2010. 35(7): p. 377-83.*

*26. Wagner, R.W., Smith, J.E., Cooperman, B.S., and Nishikura, K., A double-stranded RNA unwinding activity introduces structural alterations by means of adenosine to inosine conversions in mammalian cells and Xenopus eggs. Proc Natl Acad Sci U S A, 1989. 86(8): p. 2647-51.*

*27. Solomon, O., Di Segni, A., Cesarkas, K., Porath, H.T., Marcu-Malina, V., Mizrahi, O., Stern-Ginossar, N., Kol, N., Farage-Barhom, S., Glick-Saar, E., Lerenthal, Y., Levanon, E.Y., Amariglio, N., Unger, R., Goldstein, I., Eyal, E., and Rechavi, G., RNA editing by ADAR1 leads to context-dependent transcriptome-wide changes in RNA secondary structure. Nat Commun, 2017. 8(1): p. 1440.*

*28. Solomon, O., Segni, A.D., Cesarkas, K., Porath, H.T., Marcumalina, V., Mizrahi, O., Sternginossar, N., Kol, N., Faragebarhom, S., and Glicksaar, E.J.N.C., RNA editing by ADAR1 leads to context-dependent transcriptome-wide changes in RNA secondary structure. 2017. 8(1): p. 1440.*

*29. Castella, S., Bernard, R., Corno, M., Fradin, A., and Larcher, J.C., Ilf3 and NF90 functions in RNA biology. Wiley Interdiscip Rev RNA, 2015. 6(2): p. 243-56.*

*30. Jayachandran, U., Grey, H., and Cook, A.G., Nuclear factor 90 uses an ADAR2-like binding mode to recognize specific bases in dsRNA. Nucleic Acids Res, 2016. 44(4): p. 1924-36.*

*31. Cienikova, Z., Jayne, S., Damberger, F.F., Allain, F.H., and Maris, C., Evidence for cooperative tandem binding of hnRNP C RRMs in mRNA processing. RNA, 2015. 21(11): p. 1931-42.*

**Second round of review**

**Reviewer 1**

For major points 1 and 2, the author has provided detailed explanations and data support, which has solved my doubts. For major points 3 and 5, although the author did not provide further evidence of bioinformatics or experiments, he also introduced lots of published literature for discussion, which partially answered my questions.

Regarding major point 4, I agree that absolute numbers can reflect the potential functional associations better. But the overlaps between RBP-binding regions were quantified by the "Jaccard index" in the RBP interaction map, and the edges with a degree less than 0.1 seem not to be displayed. In conclusion, the construction standards of the two RBP interaction networks are inconsistent. The edges of one network represent the number of overlaps, and the edges of the other represent the degree of overlaps. It is recommended to unify, otherwise this conclusion (lines 42-48 on page 12) cannot be easily drawn.

In addition, I found some new questions:

1. On page 7, the author needs to check the number of "616 comparisons", there are only 614 comparisons in the supplementary Table4. Please check the number of 181 significant differences.

2. On page 9, the author seems to have updated the data in Figure 3a, please check the number of "5562 RNA editing sites" in the main text.

3. In Figure 1b, dose the "N" represents the same RNA editing sites in Figure 1a? For example, the number of RNA editing sites shared between the Nucleus and whole cell in Figure 1b is 4998, but the overlap of RNA editing sites is 5011 in Figure 1a.

4. In Figure 3a and subsequent analysis, did the authors employ only RBP-associated editing events detected from whole-cell RNA-seq and eCLIP-seq datasets?

5. Other minor errors: Unified 3' UTR writing; Correct the spelling of "supplymentaryTable" in Supplementary Files.

The article consists of extensive bioinformatics analysis, and the authors also need to double-check the data in the main text and supplementary materials.

**Authors Response**

**Point-by-point responses to the reviewers’ comments:**

*Response: We are delighted to see that our previous revision has addressed most of the reviewer’s questions. As the reviewer has suggested, we updated the RBP network in Fig. 4C to represent the overlapping regions rather than the Jaccard indices. We would like to thank the reviewer again for pointing out the inconsistent numbers and typos. We are sorry for the mistakes, and all the numbers, files, and figures have been carefully checked.*

Reviewer #1:

For major points 1 and 2, the author has provided detailed explanations and data support, which has solved my doubts. For major points 3 and 5, although the author did not provide further evidence of bioinformatics or experiments, he also introduced lots of published literature for discussion, which partially answered my questions.

Regarding major point 4, I agree that absolute numbers can reflect the potential functional associations better. But the overlaps between RBP-binding regions were quantified by the "Jaccard index" in the RBP interaction map, and the edges with a degree less than 0.1 seem not to be displayed. In conclusion, the construction standards of the two RBP interaction networks are inconsistent. The edges of one network represent the number of overlaps, and the edges of the other represent the degree of overlaps. It is recommended to unify, otherwise this conclusion (lines 42-48 on page 12) cannot be easily drawn.

*Response: We agree with the reviewer that it makes more sense to use the same standard for building the two RBP networks in Figs. 4B and 4C. Therefore, we have updated Fig. 4C and Fig. S7 with a new RBP network, in which the edges represent the overlapping RNA regions bound by two RBPs. These two RBP interaction networks indeed appear markedly different. Therefore, the main conclusion (lines 42-48 on page 12) is not affected by this modification.*

In addition, I found some new questions:

1. On page 7, the author needs to check the number of "616 comparisons", there are only 614 comparisons in the supplementary Table4. Please check the number of 181 significant differences.

*Response: We are sorry for this mistake, and we thank the reviewer for pointing this out. The numbers have been corrected. We have also carefully checked all the other numbers, files, and figures.*

2. On page 9, the author seems to have updated the data in Figure 3a, please check the number of "5562 RNA editing sites" in the main text.

*Response: We are sorry for not updating the text in the previous revision. They have been checked and corrected now.*

3. In Figure 1b, dose the "N" represents the same RNA editing sites in Figure 1a? For example, the number of RNA editing sites shared between the Nucleus and whole cell in Figure 1b is 4998, but the overlap of RNA editing sites is 5011 in Figure 1a.

*Response: We are sorry for the inconsistency when using different tools for counting the intersecting sites. We have updated Figs. 1B and S2B to correct the errors.*

4. In Figure 3a and subsequent analysis, did the authors employ only RBP-associated editing events detected from whole-cell RNA-seq and eCLIP-seq datasets?

*Response: Yes. The vast majority of the RNA editing events were detected from the RNA-seq data of the whole-cell samples (Fig. 1A), and this is at least partly due to the much higher sequencing depth for the whole-cell samples (Fig. S1C, D, Table S2). As a result, the majority of the RBP-associated RNA editing sites were identified by using the whole-cell RNA-seq data and the eCLIP-seq datasets (Fig. S5, which was Fig. S11 in the previous version). Furthermore, 50-70% of the RBP-editing associations identified with the cytosolic or nuclear fraction RNA-seq data were also found with the whole-cell RNA-seq data (Fig. S5). Therefore, Fig. 3A and the subsequent analyses were done with the sites identified from the whole-cell data. We have made this clear in the revised manuscript (page 9), and the previous Fig. S11 has been moved up as Fig. S5.*

5. Other minor errors: Unified 3' UTR writing; Correct the spelling of "supplymentaryTable" in Supplementary Files.

*Response: We thank the reviewer for pointing out these errors. They have been corrected now.*

The article consists of extensive bioinformatics analysis, and the authors also need to double-check the data in the main text and supplementary materials.

*Response: Indeed, we have carefully double-checked all the numbers and data in the main text, figures, supplementary figures, and tables.*

**Third round of review**

**Reviewer 1**

The author has solved my questions and refined the details of the manuscript.
